# The IBI1 Receptor of β-Aminobutyric Acid Interacts with VOZ Transcription Factors to Regulate Abscisic Acid Signaling and Callose-Associated Defense

**DOI:** 10.1016/j.molp.2020.07.010

**Published:** 2020-10-05

**Authors:** Roland E. Schwarzenbacher, Grace Wardell, Joost Stassen, Emily Guest, Peijun Zhang, Estrella Luna, Jurriaan Ton

**Affiliations:** 1 P3 Institute for Plant and Soil Biology, Department of Animal and Plant Sciences, The University of Sheffield, Sheffield S10 2TN, UK

**Keywords:** IBI1, priming, abscisic acid, β-aminobutyric acid, callose, E-MTAB-8720

## Abstract

External and internal signals can prime the plant immune system for a faster and/or stronger response to pathogen attack. β-aminobutyric acid (BABA) is an endogenous stress metabolite that induces broad-spectrum disease resistance in plants. BABA perception in *Arabidopsis* is mediated by the aspartyl tRNA synthetase IBI1, which activates priming of multiple immune responses, including callose-associated cell wall defenses that are under control by abscisic acid (ABA). However, the immediate signaling components after BABA perception by IBI1, as well as the regulatory role of ABA therein, remain unknown. Here, we have studied the early signaling events controlling IBI1-dependent BABA-induced resistance (BABA-IR), using untargeted transcriptome and protein interaction analyses. Transcriptome analysis revealed that IBI1-dependent expression of BABA-IR against the biotrophic oomycete *Hyaloperonospora arabidopsidis* is associated with suppression of ABA-inducible abiotic stress genes. Protein interaction studies identified the VOZ1 and VOZ2 transcription factors (TFs) as IBI1-interacting partners, which are transcriptionally induced by ABA but suppress pathogen-induced expression of ABA-dependent genes. Furthermore, we show that VOZ TFs require nuclear localization for their contribution to BABA-IR by mediating augmented expression of callose-associated defense. Collectively, our study indicates that the IBI1-VOZ signaling module channels pathogen-induced ABA signaling toward cell wall defense while simultaneously suppressing abiotic stress-responsive genes.

## Teaser

Perception of the plant stress metabolite β-aminobutyric acid (BABA) by the aspartyl tRNA synthetase IBI1 primes callose-associated cell wall defenses that are controlled by the plant hormone abscisic acid (ABA). This study investigated the early signaling events controlling IBI1-dependent BABA-induced resistance against downy mildew and identified the ABA-inducible VOZ transcription factors as downstream regulators of BABA-primed callose defense. The interaction between VOZ and IBI1 after downy mildew infection channels pathogenesis-related ABA signaling toward callose defense, while simultaneously suppressing abiotic stress-responsive genes.

## Introduction

Plants can acquire broad-spectrum disease resistance after perception of stress-indicating signals. To avoid the costs of constitutive defense expression, plants have evolved defense priming (Conrath et al., 2006; Ahmad et al., 2010), which allows for a faster and/or stronger immune response after subsequent pathogen attack (Zimmerli et al., 2000; Pastor et al., 2013). Priming can be elicited by external and internal stress stimuli, including microbe-associated molecular patterns and endogenous immune signals (Conrath et al., 2006). One metabolite that has garnered much interest over recent years is β-aminobutyric acid (BABA). Application of this non-proteinogenic amino acid primes systemic plant defenses that are controlled by both salicylic acid (SA)-dependent and SA-independent pathways, resulting in broad-spectrum resistance against biotrophic and necrotrophic pathogens (Zimmerli et al., 2000; Ton et al., 2005; Cohen et al., 2016).

While the resistance-inducing properties of BABA have been known for decades (Papavizas, 1964; Cohen et al., 2016), it was only recently discovered that this compound accumulates in stress-exposed plants (Thevenet et al., 2017), indicating that it acts as an endogenous stress metabolite (Baccelli and Mauch-Mani, 2017). This discovery is consistent with our previous finding that the aspartyl tRNA synthetase (AspRS) IBI1 in *Arabidopsis thaliana* (*Arabidopsis*) acts as an enantiomer-specific receptor of BABA (Luna et al., 2014). While the primary function of AspRS enzymes is the charging of tRNA^Asp^ with L-aspartic acid (L-Asp) for protein biosynthesis, Luna et al. (2014) showed that the biologically active R-enantiomer of BABA, which is structurally similar to L-Asp, binds *in planta* to IBI1 to activate defense priming and disease resistance. It was proposed that binding of R-BABA to the L-Asp-binding domain induces a secondary defense activity by IBI1 (Luna et al., 2014). This mode of action was recently confirmed by our finding that site-directed mutagenesis of the L-Asp binding domain of IBI1 blocks BABA-induced resistance (BABA-IR; Buswell et al., 2018).

Apart from broad-spectrum resistance, BABA also induces plant stress, which at high concentrations can result in growth reduction (Wu et al., 2010; Luna et al., 2014). This undesirable side effect has hampered commercial exploitation of BABA in crop protection schemes. It has been demonstrated in *Arabidopsis* that BABA-induced stress is caused by the inhibitory activity of R-BABA on cellular AspRS activity, leading to enhanced accumulation of uncharged tRNA^Asp^. This induces phosphorylation of the eukaryotic initiation factor eIF2α by the protein kinase GCN2, which alters cellular metabolism through translational regulation (Lageix et al., 2008), ultimately resulting in a plant stress response that is associated with growth inhibition. Since the *gcn2-1* mutant was found to be more tolerant to BABA-induced stress but unaffected in BABA-IR, it was concluded that the GCN2-dependent pathway does not contribute to BABA-IR (Luna et al., 2014). Conversely, mutants in *IBI1* were not only impaired in BABA-IR but were also hypersensitive to BABA-induced stress. This contrasting response to BABA was explained by the fact that *ibi1* mutants have strongly reduced AspRS activity, which makes them more prone to BABA-induced accumulation of uncharged tRNA^Asp^ and GCN2-dependent stress (Luna et al., 2014). Hence, IBI1 controls BABA-IR and BABA-induced stress via separate pathways.

Compared with the GCN2-dependent pathway controlling BABA-induced stress, less is known about the early signaling steps controlling IBI1-dependent BABA-IR. While it is known that BABA primes multiple immune responses that become active at different stages of pathogen infection, there is no direct mechanistic link between IBI1 perception of BABA and defense priming. In addition to relatively late-acting SA-dependent defense mechanisms (Zimmerli et al., 2000; Ton et al., 2005; Van der Ent et al., 2009), BABA also primes early-acting cell wall defenses, which are under control by abscisic acid (ABA) (Ton and Mauch-Mani, 2004; Flors et al., 2008; Pastor et al., 2013). However, how BABA-activated IBI1 regulates ABA and SA signaling to mediate augmented defense induction after pathogen attack remains unclear. While there is ample evidence that ABA suppresses SA-dependent immunity (Audenaert et al., 2002; Mohr and Cahill, 2003; Anderson et al., 2004; de Torres-Zabala et al., 2007; Yasuda et al., 2008; Fan et al., 2009; Ding et al., 2016; Berens et al., 2019), other studies have demonstrated a positive role for ABA in plant immunity, particularly with regard to callose-associated cell wall defense (Ton and Mauch-Mani, 2004; Ton et al., 2005; Adie et al., 2007; García-Andrade et al., 2011; Oide et al., 2013; Hok et al., 2014). Furthermore, previous studies of BABA-IR have reported changes at the transcriptional, post-translational, and metabolic level in response to BABA treatment alone (Van der Ent et al., 2009; Conrath, 2011; Bengtsson et al., 2014; Pastor et al., 2014), but it remains unclear whether these changes contribute to BABA-IR or whether they are the result of BABA-induced stress. One clue about the early signaling mechanisms by which IBI1 controls BABA-IR comes from its subcellular localization (Schwarzenbacher et al., 2014). In unstressed plants, IBI1 is associated with the endoplasmic reticulum (ER), which remains unaltered after BABA treatment alone, indicating that the subcellular localization of IBI1 is not affected by BABA-induced stress (Luna et al., 2014). However, pathogen infection triggers a partial translocation of IBI1 from the ER to the cytoplasm, which is strongly augmented in BABA-primed plants. Thus, the cytoplasmic localization of IBI1 correlates with the augmented defense expression during BABA-IR, indicating that binding of BABA to IBI1 primes the protein for pathogen-induced translocation to the cytoplasm, where it initiates defense expression through interaction with yet unknown defense-regulatory proteins.

Here, we have investigated the link between IBI1 and BABA-IR, using a combination of untargeted approaches. Global transcriptome analysis of wild-type and *ibi1-1 Arabidopsis* in the presence and absence of the biotrophic pathogen *Hyaloperonospora arabidopsidis* (*Hpa*) enabled us to separate stress-related gene expression from defense-related gene expression, revealing that IBI1-dependent BABA-IR is associated with repression of ABA-dependent gene expression. In addition, yeast two-hybrid (Y2H) analysis identified the NAC gene family transcription factors (TFs) Vascular Plant One Zinc Finger 1 (VOZ1) and VOZ2 as interactors of IBI1. We show that these two ABA-responsive TFs are responsible for the repression of ABA-inducible gene expression during BABA-IR against *Hpa*. Furthermore, we provide evidence that the nuclear activity of these TFs controls BABA-IR through priming of predominantly callose-associated defenses at the cell wall. Based on these results, we present a model in which the IBI1-VOZ signaling module acts as a switch that channels pathogen-induced ABA signaling toward cell wall defense while repressing induction of ABA-responsive abiotic stress genes, thereby reconciling previous contradictory reports about the role of ABA in plant immunity.

## Results

### IBI1 Regulates Global Gene Responses to BABA

**Figure 1:**
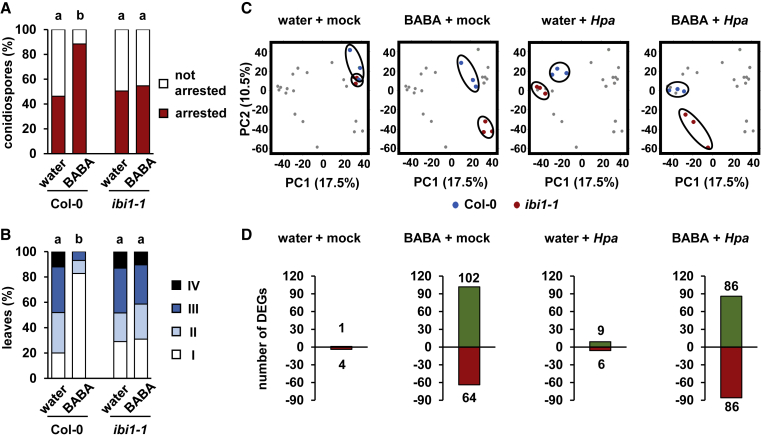
Global Transcriptome Analysis of Wild-Type *Arabidopsis* (Col-0) and the BABA Receptor Mutant *ibi1-1*. Three-week-old plants were soil-drenched with water or BABA (10 mg/l) and challenged with conidiospores of *Hyaloperonospora arabidopsidis* (*Hpa*) 2 days later. Replicate shoot samples for RNA extraction and analysis of callose deposition were collected at 2 dpi. Samples for the analysis of *Hpa* colonization were collected at 5 dpi. **(A)** Quantification of the effectiveness of callose depositions in arresting *Hpa* colonization at the epidermal cell layer. *Hpa*-induced callose was analyzed in aniline blue/calcofluor-stained leaves by epifluorescence microscopy. Shown are percentages of callose-arrested and non-arrested conidiospores (see Supplemental Figure 7 for details). Different letters indicate statistically significant differences in class distribution between samples (Fisher's exact tests + FDR; *p* < 0.05; *n* = 110–130 conidiospores). **(B)** BABA-induced resistance (BABA-IR) against *Hpa*. Pathogen colonization was determined by microscopy analysis of trypan blue-stained leaves. Leaves were assigned to different *Hpa* colonization classes (I: no growth; II: hyphal growth; III: hyphal growth + conidiophores; IV: extensive hyphal growth + conidiophores + oospores; see Supplemental Figure 6 for details). Different letters indicate statistically significant differences in class distribution between samples (Fisher's exact tests + FDR; *p* < 0.05; *n* = 25–30 leaves). **(C)** Principal component analysis (PCA) of relative gene expression values (Arabidopsis Gene ST 1.1 array). Biologically replicated samples (*n* = 3) from Col-0 (blue) and *ibi1-1* (red) are highlighted within the same PCA plot for each of the four experimental conditions (water + mock, BABA + mock, water + *Hpa*, and BABA + *Hpa*). **(D)** Numbers of differentially expressed genes (DEGs) between Col-0 and *ibi1-1* under the four different (pre-)treatment conditions (linear model + FDR; *q* < 0.01). Red bars indicate downregulated DEGs in *ibi1-1*; green bars indicate upregulated DEGs in *ibi1-1*.

To obtain a global impression of IBI1-dependent gene expression during the expression of BABA-IR, we analyzed the transcriptome of water (control)- and BABA-treated Columbia-0 (Col-0; wild-type) and *ibi1-1* mutant plants after challenge inoculation with water (mock) or *Hpa* conidiospores. Three biological replicates per treatment–genotype combination were collected at 2 days post inoculation (dpi), which represents an early time point in the interaction, when *Hpa* conidiospores are starting to penetrate the epidermal cell layer. To verify a primed immune response at this stage, we analyzed *Hpa*-inoculated leaves by epifluorescence microscopy to determine the percentage of callose-rich papillae arresting *Hpa* germ tubes. As expected, BABA-treated Col-0 plants displayed a statistically significant increase in the percentage of callose-arrested *Hpa* germ tubes compared with water-treated Col-0, whereas the *ibi1-1* mutant failed to express this augmented cell wall defense (Figure 1A). These results were consistent with the extent of *Hpa* colonization at 5 dpi in trypan blue-stained leaves, confirming that BABA-treated Col-0 plants, unlike the *ibi1-1* mutant, showed a strong and statistically significant reduction in *Hpa* colonization (Figure 1B).

Transcriptome analysis was based on the Arabidopsis Gene 1.1 ST array (Affymetrix), which contains 600 941 gene probes of 28 501 genes. To verify that the experimental design of our transcriptome experiment was suitable to detect a global response to *Hpa*, we first analyzed the differences in gene expression between mock- and *Hpa*-inoculated wild-type plants that had not been treated with BABA. This analysis identified 477 differentially expressed genes (DEGs), of which 446 showed >2-fold induction by *Hpa* (Supplemental Table 1). Such response is comparable with previous transcriptome studies of *Hpa*-infected *Arabidopsis* (Huibers et al., 2009; López Sánchez et al., 2016), thus confirming that the timing of sample collection and general conditions of our experiment were suitable for detection of a transcriptional host response to *Hpa*.

We next investigated the global impact of IBI1 on BABA- and *Hpa*-inducible defense responses. Unsupervised principal component analysis (PCA) revealed clustering of samples according to treatments and genotypes (Figure 1C). To visualize the contribution of IBI1 under the different experimental conditions, we highlighted samples from similarly treated Col-0 and *ibi1-1* plants within the same PCA plot across all four conditions (Figure 1C). Differences between Col-0 and *ibi1-1* were most pronounced after pre-treatment with BABA, irrespective of the secondary challenge treatment, indicating that IBI1 plays a major regulatory role in the transcriptomic response to BABA.

### Comparative Transcriptome Analysis Separates Stress-Related Gene Expression from Defense-Related Gene Expression during BABA-IR

To identify the genes that are under transcriptional control by IBI1, we selected differentially expressed genes (DEGs) between Col-0 and *ibi1-1* under each of the four experimental conditions (i.e., water + mock, BABA + mock, water + *Hpa*, and BABA + *Hpa*). Under stress-free conditions (water + mock), there were only five DEGs between Col-0 and *ibi1-1* (Figure 1D and Supplemental Table 2). This low number of differential gene expression is consistent with the lack of developmental growth phenotypes of *ibi1-1*. Similarly, we only detected 15 DEGs in unprimed plants after *Hpa* inoculation, supporting our observations that *ibi1-1* is not majorly affected in basal resistance against *Hpa* (Luna et al., 2014; Figure 1D). By contrast, we detected 166 DEGs between Col-0 and *ibi1-1* after BABA + mock treatment, and 172 DEGs after BABA + *Hpa* treatment (Figure 1D and Supplemental Table 2), confirming that IBI1 controls a relatively large set of BABA-responsive genes.

**Figure 2:**
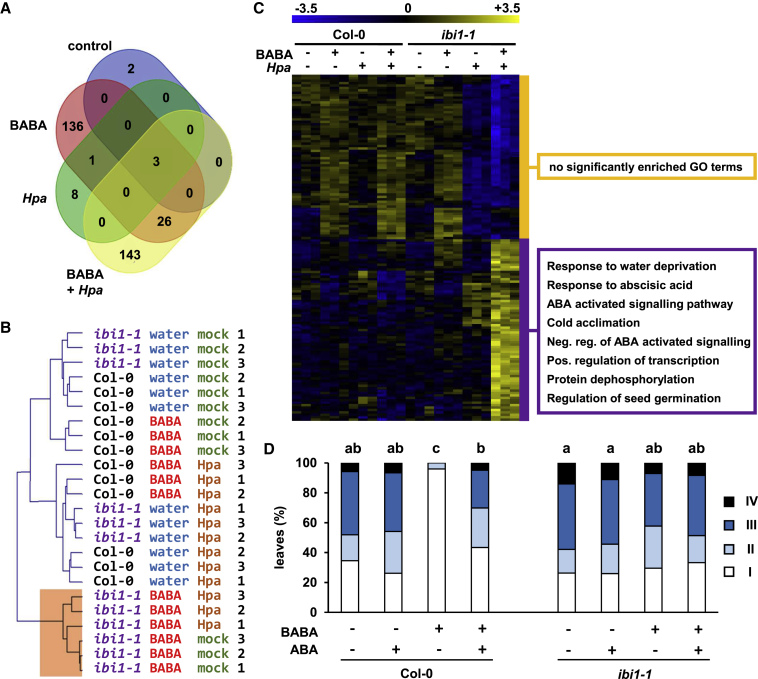
Analysis of IBI1-Dependent Genes Associated with BABA-Augmented Defense against *Hpa* Reveals a Role for Suppression of ABA-Dependent Signaling. **(A)** Venn diagram of DEGs between Col-0 and *ibi1-1* under the four different experimental conditions. **(B)** Hierarchical clustering of all samples according to the 136 DEGs that respond to BABA + mock treatment only. Samples from all BABA-treated *ibi1-1* plants (BABA + mock and BABA + *Hpa*) cluster apart from samples of other treatment/genotype combinations. Accordingly, these 136 DEGs correlate with differences in BABA-induced stress rather than BABA-augmented defense expression after *Hpa* inoculation. Numbers on the right denote biological replicates of each genotype-treatment combination. **(C)** Hierarchical clustering and expression levels of 143 DEGs that respond to BABA + *Hpa* treatment and not BABA + mock treatment. Accordingly, these 143 DEGs correlate with BABA-augmented defense expression against *Hpa* but not BABA-induced stress. Colored boxes on the right indicate GO terms that are statistically significantly enriched within each gene cluster. **(D)** ABA application suppresses BABA-IR against *Hpa*. Two-week-old Col-0 and *ibi1-1* plants were treated with 5 mg/l BABA or water. At 24 h after BABA treatment, plants were sprayed with ABA (10 μM + 0.01% Silwet) or mock solution (0.5% EtOH + 0.01% Silwet). At 48 h after BABA treatment, plants were challenged with *Hpa*. Disease progression was assessed at 5 dpi by assigning trypan blue-stained leaves to four different colonization classes (Supplemental Figure 6). Different letters indicate statistically significant differences in class distribution between samples (Fisher's exact tests + FDR; *p* < 0.05; *n* = 52–83 leaves).

Since the *ibi1-1* mutant is not only impaired in BABA-IR but is also hypersensitive to BABA-induced stress (Luna et al., 2014; Buswell et al., 2018), the DEGs after BABA treatment can be related to either BABA-induced stress or BABA-augmented defense against *Hpa*. To separate the stress-related genes from the defense-related genes, we selected the 136 DEGs that are unique to BABA + mock treatment (Figure 2A), during which Col-0 and *ibi1-1* develop different levels of BABA-induced stress but do not express different levels of *Hpa*-induced defense. Hierarchical clustering by the expression profiles of these 136 genes revealed that samples from BABA-treated *ibi1-1* plants clustered apart from all other samples, irrespective of secondary inoculation treatment (mock or *Hpa*; Figure 2B). By contrast, samples from water- and BABA-treated Col-0 after *Hpa* inoculation (BABA + *Hpa* and water + *Hpa*) clustered closely to those from water-treated *ibi1-1* plants after *Hpa* inoculation (water + *Hpa*; Figure 2B). Thus, the 136 DEGs responding to BABA + mock treatment only show similar expression patterns in both resistant and susceptible plants, indicating that these genes are unrelated to plant resistance, but rather mark the genotypic differences in BABA-induced stress tolerance. Therefore, to select for genes that are related to BABA-augmented defense against *Hpa*, we removed all stress-related genes from the group of 172 DEGs after BABA + *Hpa* treatment. The resulting 143 DEGs, which are unique for the BABA + *Hpa* condition (Figure 2A), represent IBI1-controlled genes that are associated with BABA-augmented defense expression against *Hpa*.

### BABA-IR against *Hpa* Is Associated with IBI1-Dependent Suppression of ABA-Dependent Signaling

Hierarchical clustering of the 143 defense-related DEGs revealed two distinct gene clusters with opposite expression patterns between Col-0 and *ibi1-1* after BABA + *Hpa* treatment (Figure 2C and Supplemental Table 2B). Statistical gene ontology (GO)-term enrichment analysis revealed that the cluster displaying enhanced expression in *ibi1-1* compared with Col-0 is strongly enriched with genes related to ABA signaling and abiotic stress tolerance (Figure 2C). By contrast, there was no enrichment for any GO terms among the 136 genes that were differentially expressed in response to BABA only. Hence, IBI1-dependent repression of ABA-inducible genes is associated with BABA-IR against *Hpa* and not BABA-induced stress. To examine the significance of ABA signaling in BABA-IR against *Hpa*, we tested the effects of co-application of BABA with ABA on the level of BABA-IR. To this end, 2-week-old Col-0 and *ibi1-1* plants were soil-drenched with BABA or water, sprayed with 10 μM ABA or mock solution at 1 day after BABA treatment, and challenged with *Hpa* at 2 days after BABA treatment. Analysis of *Hpa* colonization at 5 dpi revealed that ABA treatment completely repressed BABA-IR in Col-0 against *Hpa* (Figure 2D). Similar results were obtained with a 10-fold higher concentration of ABA (100 μM; Supplemental Figure 1A). Furthermore, reversing the order of ABA and BABA application did not change the outcome of this experiment (Supplemental Figure 1B). Hence, ABA represses BABA-IR against *Hpa*. Together with the results of our transcriptome analysis, this strongly indicates that BABA-IR against *Hpa* requires IBI1-dependent repression of ABA signaling.

### IBI1 Interacts with the Transcription Factors VOZ1 and VOZ2

**Figure 3:**
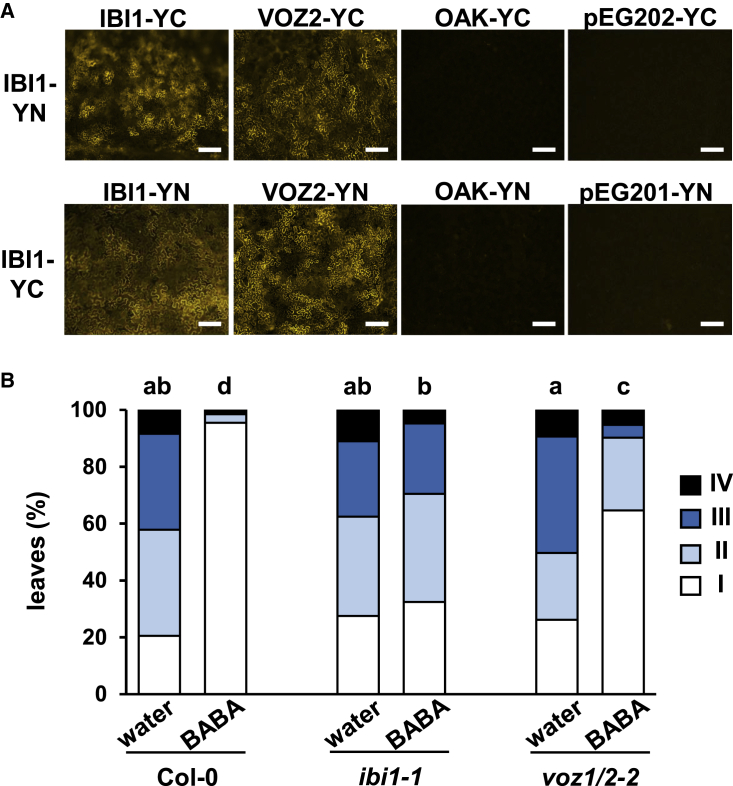
VOZ Proteins Interact with IBI1 *In Planta* and Are Required for BABA-IR. **(A)** The interaction between IBI1 and VOZ2 was tested by bimolecular fluorescence complementation (BiFC) analysis. Full-length gene constructs encoding proteins with a C-terminal fusion of the amino-terminal (-YN) or carboxyl-terminal (-YC) half of YFP were transiently expressed in *N*. *benthamiana* via leaf infiltration with *A*. *tumefaciens*. Specific interactions between protein constructs were determined by epifluorescence microscopy of YFP fluorescence at 3 days after infiltration. Co-infiltration with YN- and YC-tagged IBI1 proteins was used as a positive control to visualize the IBI1 autointeraction. Co-infiltrations of YN/YC-tagged IBI1 protein with the YC/YN-tagged membrane protein OAK or the empty vectors (pEarleygate201/202) were used as negative controls. Scale bars, 200 μm. **(B)** The *voz1-2*/*voz2-2* double mutant (*voz1*/*2-2*) is partially compromised in BABA-IR against *Hpa*. Two-week-old Col-0, *ibi1-1*,and *voz1*/*2-2* plants were soil-drenched with water or 5 mg/l BABA and inoculated with *Hpa* conidiospores 2 days later. Disease progression was determined at 5 dpi by assigning trypan blue-stained leaves to different *Hpa* colonization classes (Supplemental Figure 6). Different letters indicate statistically significant differences in class distribution between samples (Fisher's exact tests + FDR; *p* < 0.05; *n* = 60–150 leaves).

Based on our previous finding that IBI1 moves from the ER to the cytoplasm during BABA-IR against *Hpa* (Luna et al., 2014), we hypothesized that the increased pool of cytoplasmic IBI1 mediates the BABA-augmented defense response by interacting with defense-regulatory proteins. To identify such IBI1-interacting proteins, we screened two different cDNA libraries of *Arabidopsis* by Y2H analysis, using the full-length IBI1 protein as a bait. The first screen was based on a cDNA library from 1-week-old *Arabidopsis* seedlings while the second screen used a cDNA library from a mixture of *Arabidopsis* tissues at different developmental stages. Together, both screens yielded 25 putative interactors (Supplemental Figure 2A and Supplemental Table 3). Since BABA-IR against *Hpa* manifests itself in the leaves, we first selected candidates that are expressed in leaves, using publicly available gene expression data from the eFP Browser (Schmid et al., 2005; Winter et al., 2007; Klepikova et al., 2016). From the remaining 15 candidates, we selected three high-confidence interactors that had previously been shown to localize to the cytoplasm (Hooper et al., 2017), which is the subcellular location of IBI1 during expression of BABA-augmented defense against *Hpa*. Apart from IBI1 itself, we identified the VOZ TFs VOZ1 and VOZ2 (Supplemental Figure 2B), which are functionally redundant to each other (Yasui et al., 2012; Nakai et al., 2013). *In planta* interactions of IBI1–IBI1 and IBI1–VOZ2 were confirmed using bimolecular fluorescence complementation (BiFC) assays. Co-infiltration of *Nicotiana benthamiana* leaves with *Agrobacterium tumefaciens* strains expressing complementary IBI1-YFP (yellow fluorescent protein) constructs (IBI1 autointeraction), as well as complementary IBI1-YFP and VOZ2-YFP constructs (IBI1–VOZ2 interaction), elicited a strong YFP fluorescence signal for each reciprocal combination (Figure 3A). By contrast, the four negative controls (IBI1-YFP constructs co-infiltrated with the empty vector strains and IBI1-YFP constructs co-infiltrated with complementary YFP fusion constructs of the protein kinase OAK) failed to elicit YFP fluorescence.

### Mutations in *VOZ1* and *VOZ2* Attenuate BABA-IR against *Hpa*

The VOZ1 and VOZ2 TFs have previously been reported to repress ABA-dependent gene expression and abiotic stress tolerance (Nakai et al., 2013). Since our transcriptome analysis had revealed that BABA-IR against *Hpa* is associated with IBI1-dependent suppression of ABA-dependent abiotic stress genes (Figure 2), we investigated the role of VOZ1 and VOZ2 in BABA-IR against *Hpa*. Because both *VOZ* genes are functionally redundant to each other (Yasui et al., 2012; Nakai et al., 2013), we tested BABA-IR in the *voz1-2*/*voz2-2* double mutant (*voz1*/*2-2*) and compared its resistance phenotype with Col-0 and *ibi1-1*. As expected, BABA-treated Col-0 plants showed almost complete protection against *Hpa*, whereas *ibi1-1* failed to express BABA-IR (Figure 3B). Notably, while BABA-treated *voz1*/*2-2* still expressed a statistically significant resistance response to *Hpa*, the level of BABA-IR was attenuated compared with the wild type, as evidenced by a statistically higher level of *Hpa* colonization in BABA-treated *voz1*/*2-2* compared with BABA-treated Col-0. Hence, the IBI1-interacting VOZ proteins contribute to BABA-IR against *Hpa*.

### The *voz1*/*2-2* Mutant Is Affected in Priming of SA-Induced Gene Expression

**Figure 4:**
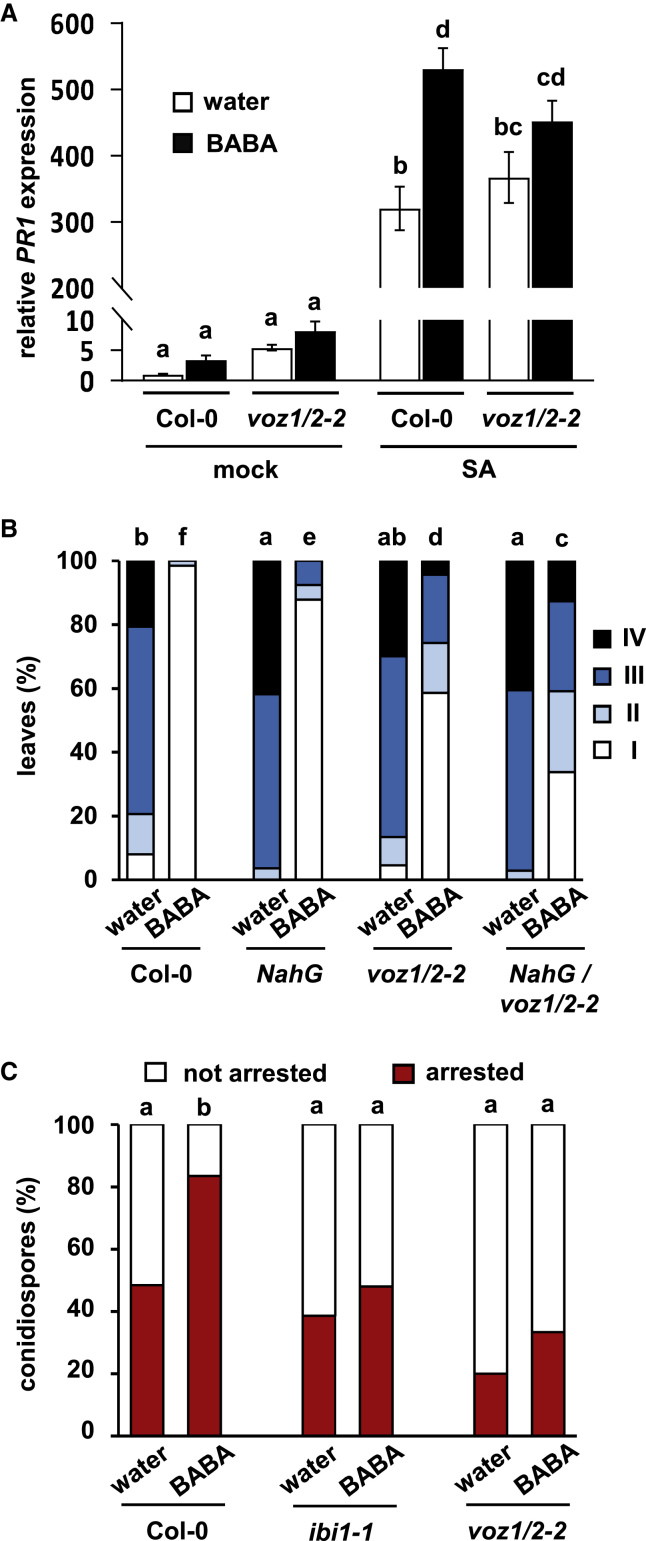
The Signaling Role of VOZ1 and VOZ2 in BABA-IR. **(A)** The *voz1*/*2-2* mutant is partially compromised in BABA-induced priming of salicylic acid (SA)-induced *PR1* expression. Leaves of water- and BABA-treated Col-0 and *voz1*/*2-2* were sprayed with 1 mM SA at 2 days after soil-drench treatment of 2-week-old plants with water or BABA (5 mg/l). Shoots were harvested at 8 h after SA treatment for qRT–PCR analysis. Data shown are mean expression values (±SEM) of *PR1* expression relative to water-treated mock-sprayed Col-0 plants. Different letters indicate statistically significant differences in expression (two-way ANOVA + Tukey's multiple comparisons test; *p* < 0.05; *n* = 4). **(B)** Introgression of the *NahG* gene in the *voz1*/*2-2* mutant background further reduces its attenuated BABA-IR response, indicating that SA-dependent defense does not play a major role in VOZ1/2-dependent BABA-IR against *Hpa*. Two-week-old Col-0, *NahG*, *voz1*/*2-2*, and *NahG voz1*/*2-2* plants were soil-drenched with water or 5 mg/l BABA and inoculated with *Hpa* 2 days later. Disease progression was assessed at 5 dpi by assigning trypan blue-stained leaves to different *Hpa* colonization classes (Supplemental Figure 6). Different letters indicate statistically significant differences in class distribution between samples (Fisher's exact tests + FDR; *p* < 0.05; *n* = 55–71 leaves). **(C)** The *voz1*/*2-2* mutant is impaired in BABA-induced priming of callose-associated cell wall defense. Two-week-old Col-0, *ibi1-1*, and *voz1*/*2-2* plants were soil-drenched with water or 5 mg/l BABA and inoculated with *Hpa* 2 days later. Callose effectiveness was analyzed at 3 dpi in aniline blue/calcofluor-stained leaves by epifluorescence microscopy. Shown are percentages of callose-arrested and non-arrested germ tubes at the epidermal cell layer (Supplemental Figure 7). Letters indicate statistically significant differences between samples (Fisher's exact tests + FDR; *p* < 0.05; *n* = 31–105 conidiospores).

BABA-IR against *Hpa* is based on priming of both SA-dependent and SA-independent defenses (Ton et al., 2005). Since the *voz1*/*2-2* mutant was only partly impaired in BABA-IR (Figure 3B), we hypothesized that one of these mechanisms is affected. Since the *voz1*/*2-2* mutant had previously been reported to be compromised in systemic acquired resistance (SAR) against *Pseudomonas syringae* (Nakai et al., 2013), which is predominantly based on priming of SA-dependent defenses (Kohler et al., 2002), we examined BABA-induced priming of the SA-inducible *PR1* gene in Col-0 and *voz1*/*2-2* plants. To this end, replicate leaf samples were collected from water- and BABA-treated plants at 8 h after challenge treatment with SA. In water-treated (unprimed) plants, the level of *PR1* induction by SA was similar in both genotypes (Figure 4A), indicating that *voz1*/*2-2* is unaffected in basal SA sensitivity. Furthermore, BABA-treated Col-0 displayed statistically increased levels of SA-induced *PR1* expression compared with water-treated Col-0 plants, confirming that BABA primes SA-induced gene expression (Zimmerli et al., 2000; Ton et al., 2005). While SA-induced *PR1* induction in BABA-treated *voz1*/*2-2* plants was marginally enhanced compared with water-treated *voz1*/*2-2* plants, this effect was not statistically significant (Figure 4A). Hence, the *voz1*/*2-2* mutant is partially affected in the priming of SA-induced *PR1* expression by BABA.

To determine the impact of the attenuated priming of SA-induced gene expression in *voz1*/*2-2* on BABA-IR against *Hpa*, we introgressed the *NahG* gene into the *voz1*/*2-2* background, which prevents endogenous SA accumulation (Gaffney et al., 1993; Delaney et al., 1994). If the reduced BABA-IR response of *voz1*/*2-2* was caused by its defect in priming of SA-defense gene expression (Figure 4A), introgression of *NahG* in *voz1*/*2-2* would not further compromise BABA-IR. Accordingly, we compared levels of BABA-IR against *Hpa* in Col-0, *NahG*, *voz1*/*2-2*, and *NahG voz1*/*2-*2. As reported previously (Zimmerli et al., 2000; Ton et al., 2005), BABA-IR against *Hpa* in *NahG* plants is largely unaffected, due to earlier-acting cell wall defenses that operate independently of SA (Figure 4B). While the *voz1*/*2* mutant showed attenuated BABA-IR against *Hpa* compared with Col-0 and *NahG* plants, *NahG voz1*/*2-2* plants displayed an even stronger reduction in BABA-IR, which was statistically significant (Figure 4B). These results indicate that the attenuated BABA-IR response of *voz1*/*2* is not due to its impaired priming of SA-inducible gene expression but rather due to a defect in other, SA-independent, resistance mechanisms.

### VOZ1 and VOZ2 Contribute to BABA-IR against *Hpa* by Mediating Augmented Expression of Callose-Associated Cell Wall Defense

Since priming of SA-dependent defenses did not have a major contribution to VOZ1/2-dependent BABA-IR against *Hpa* (Figure 4B), we investigated the involvement of SA-independent cell wall defenses. To this end, the effectiveness of callose depositions in water- and BABA-treated Col-0 and *voz1*/*2-2* was determined at 3 days after *Hpa* inoculation. Epifluorescence microscopy analysis of the percentage of callose-arrested germ tubes confirmed that BABA-treated Col-0 plants show a statistically significant augmentation in callose-associated defense against *Hpa* (Figure 4C). By contrast, BABA-treated *voz1*/*2-2* plants, like *ibi1-1* plants, failed to express this augmented cell wall defense (Figure 4C). Hence, the *voz1*/*2-2* mutant is impaired in BABA-induced priming of callose-associated cell wall defense against *Hpa*.

### VOZ2 Must Locate to the Nucleus for Its Contribution to BABA-IR against *Hpa*

**Figure 5:**
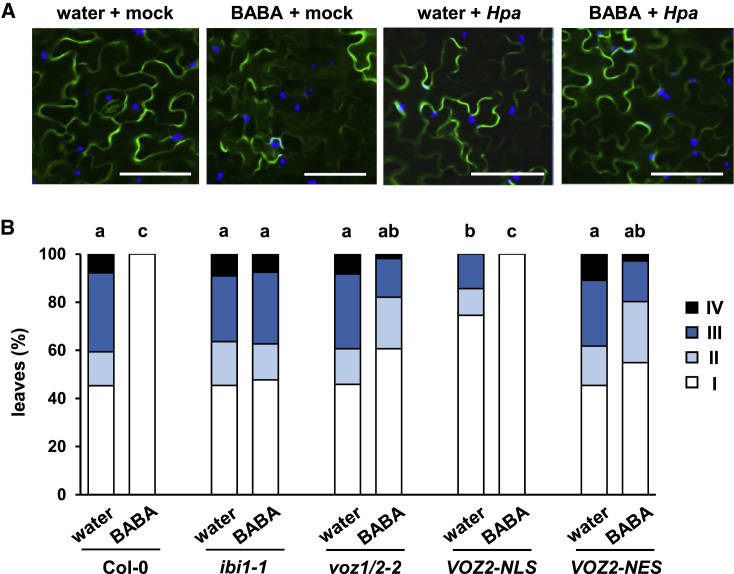
VOZ2 Is Predominantly Localized in the Cytoplasm but Requires Nuclear Localization for Its Contribution to BABA-IR against *Hpa*. **(A)** Epifluorescence microscopy of epidermal leaf cells reveals that GFP-VOZ2 is predominantly localized in the cytoplasm under all four experimental conditions, rather than the nucleus. Shown are merged fluorescence signals from DAPI-stained nuclei (blue) and GFP-VOZ2 (green). Two-week-old *voz1*/*2-1 35S*:*GFP-VOZ2* plants were soil-drenched with 5 mg/l BABA or water and challenge-inoculated with *Hpa* or water (mock) 2 days later. Samples for DAPI staining and epifluorescence microscopy were taken at 2 dpi. Scale bars, 50 μm. **(B)** BABA-IR against *Hpa* requires nuclear localization of VOZ2. Two-week-old Col-0, *voz1*/*2-2*, and *voz1*/*2-1* plants complemented with VOZ2 fused to a nuclear localization signal (*VOZ2-NLS*) or VOZ2 fused to nuclear exportation sequence (*VOZ2-NES*), were soil-drenched with water or 5 mg/l BABA and inoculated with *Hpa* 2 days later. Disease progression was analyzed in trypan blue-stained leaves at 5 dpi by assigning leaves to different *Hpa* colonization classes (Supplemental Figure 6). Different letters indicate significant differences in class distributions (Fisher's exact tests + FDR; *p* < 0.05; *n* = 60–150 leaves).

Pathogen-induced callose deposition requires activity by the callose synthase *PMR4* in *Arabidopsis* (Nishimura et al., 2003), which is regulated at the post-translational level (Flors et al., 2008; Ellinger et al., 2013; Ellinger and Voigt, 2014). Accordingly, it has been proposed that this defense layer is regulated independently of gene transcription (Van der Ent et al., 2009), which is difficult to reconcile with our finding that VOZ TFs regulate this defense (Figure 4C). We therefore investigated whether the role of VOZ2 in BABA-IR depends on its nuclear localization as a TF or whether this involves an alternative cytoplasmic function. To this end, we first characterized the subcellular localization of VOZ2 in the *voz1*/*2-1* double mutant expressing *GFP*-*VOZ2* (*p35S*:*GFP-VOZ2*; Yasui et al., 2012) under all four experimental conditions (water + mock, BABA + mock, water + *Hpa*, and BABA + *Hpa*). Even though *p35S*:*GFP-VOZ2* plants expressed wild-type levels of BABA-IR (Supplemental Figure 3A), epifluorescence microscopy of 4′,6-diamidino-2-phenylindole (DAPI)-stained leaves only revealed cytoplasmic localization of GFP-VOZ2 and no detectable co-localization with the nucleus (Figure 5A). In an independent experiment, confocal laser scanning microscopy did not detect noticeable changes in cytoplasmic localization of GFP-VOZ2 between the four different experimental conditions (Supplemental Figure 3B). However, as has been reported previously (Yasui et al., 2012), the amount of active GFP-VOZ2 in the nucleus may be too low for nuclear detection by microscopy. To examine the role of potentially low amounts of VOZ2 in the nucleus, we quantified BABA-IR in *voz1*/*2-1* plants overexpressing *VOZ2* fused to either a nuclear localization sequence (*VOZ2-NLS*) or a nuclear export sequence (*VOZ2-NES*, Yasui et al., 2012). *VOZ2-NLS-*expressing plants showed wild-type levels of BABA-IR against *Hpa* and also displayed increased basal resistance compared with water-treated Col-0 plants (Figure 5B). By contrast, *VOZ2-NES* plants showed attenuation in BABA-IR similar to that of *voz1*/*2-2* plants and showed wild-type levels of basal resistance (Figure 5B). Thus, despite the fact that we only detected GFP-VOZ2 fluorescence in the cytoplasm, nuclear localization of VOZ2 is critical for its contribution to BABA-IR against *Hpa*.

### *VOZ1*/*2* Are Transcriptionally Induced by ABA but Repress *Hpa*-Induced ABA Abiotic Stress Signaling during BABA-IR

**Figure 6:**
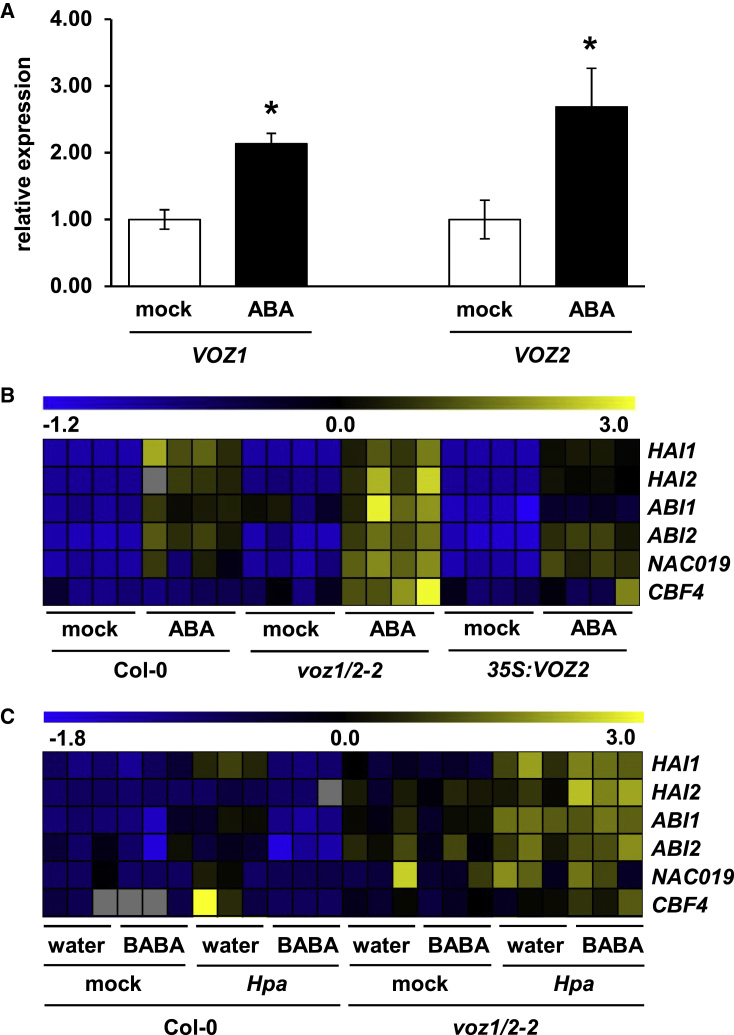
*VOZ1* and *VOZ2* Are Transcriptionally Inducible by ABA but Repress *Hpa*-Induced ABA Signaling during BABA-IR. **(A)** The *VOZ1* and *VOZ2* genes are transcriptionally induced by shoot application of abscisic acid (ABA). Two-week-old plants were sprayed with a 0.01% Silwet +0.5% EtOH solution (mock) or 0.01% Silwet +100 μM ABA. Shoot tissues were collected for qRT–PCR analysis at 8 h after treatment. Data represent mean expression values (±SEM) relative to mock-treated Col-0 plants. Asterisks indicate statistically significant differences between control and ABA treatments (Student's *t*-test; *p* < 0.05; *n* = 4). **(B)** VOZ1/2 repress ABA-inducible genes marking the abiotic stress response. Two-week-old Col-0, *voz1*/*2-2*,and *35S*:*GFP-VOZ2* plants were sprayed with 0.01% Silwet + 0.5% EtOH (mock) or 0.01% Silwet + 100 μM ABA. Replicate shoot samples for qRT–PCR analysis were collected 1 day after ABA treatment (*n* = 4). Heatmap projections show expression values of six ABA-inducible marker genes, row-normalized to the average expression value of mock-treated Col-0 samples. Gray cells indicate missing values. **(C)** VOZ1/2 repress the ABA-dependent abiotic stress response during BABA-IR against *Hpa*. Two-week-old Col-0 and *voz1*/*2-2* plants were soil-drenched with water or BABA (5 mg/l) and challenge-inoculated with mock or *Hpa* 2 days later. Replicate shoot samples for qRT–PCR analysis (*n* = 3) were collected at 4 dpi. See legend of **(B)** for details.

To investigate the signals controlling *VOZ* gene expression, we first consulted publicly available transcriptome data (eFP Browser; Winter et al., 2007). While none of the *VOZ* genes showed transcriptional induction by SA, *VOZ1* was transcriptionally induced by ABA, and both TF genes showed transient induction by salt, drought, and osmotic stress. To verify this effect of ABA on *VOZ* gene expression under our experimental growth conditions, we sprayed shoots of 2-week-old plants with 100 μM ABA or control solution (0.5% EtOH + 0.01% Silwet) and collected replicate samples 8 h later for qRT–PCR analysis. Both *VOZ1* and *VOZ2* showed statistically significant levels of induction by ABA (Figure 6A). Since VOZ1 and VOZ2 have been reported to suppress ABA-inducible genes during abiotic stress exposure (Nakai et al., 2013), this indicates that both TFs act in a negative signaling loop to control excessive induction of genes controlling abiotic stress tolerance.

Our transcriptome analysis had shown that BABA-IR against *Hpa* involves IBI1-dependent repression of ABA-inducible genes (Figures 2C and 2D). Accordingly, we hypothesized that VOZ1 and VOZ2 mediate augmented cell wall defense during BABA-IR by repressing ABA-dependent defense against abiotic stress, which is known to antagonize plant immunity (Yasuda et al., 2008; Bostock et al., 2014; Berens et al., 2019). To investigate this hypothesis, we profiled the transcription of six ABA-responsive genes in Col-0, *voz1*/*2-2*, and *35S*:*VOZ2* plants at 24 h after shoot treatment with control solution or 100 μM ABA. This gene set included genes showing IBI1-dependent repression during BABA-IR against *Hpa* (*HAI1*, *HAI2*, *ABI1*, *NAC019*; Figure 2C and Supplemental Table 2), the ABA-inducible marker gene *ABI2*, and the ABA-dependent TF gene *CBF4*, which had previously been reported to be repressed by VOZ1/2 (Nakai et al., 2013). As expected, qRT–PCR profiling of replicate samples showed increased induction of these ABA marker genes in *voz1*/*2-2* and reduced induction in *35S*:*VOZ2* compared with Col-0 plants (Figure 6B). Although we did not detect a statistically significant induction of the *VOZ1* and *VOZ2* genes by *Hpa* at the time points of our qRT–PCR experiments (data not shown), mining of publicly available transcriptome data from a time-course experiment by Wang et al. (2011) revealed that infection by virulent *Hpa* transiently induces both *VOZ1* and *VOZ2* (Supplemental Figure 4). To further test our hypothesis that VOZ1 and VOZ2 repress ABA-dependent abiotic stress signaling during BABA-IR against *Hpa*, we profiled the expression of the six ABA marker genes in water- and BABA-pre-treated Col-0 and *voz1*/*2-2* after mock or *Hpa* inoculation. At 4 dpi, the ABA-responsive genes showed transcriptional induction by *Hpa*, which was stronger in *voz1*/*2-2* plants (Figure 6C). Moreover, BABA pre-treatment completely prevented the induction of ABA marker genes by *Hpa* in Col-0 but not *voz1*/*2-2* (Figure 6C). Hence, VOZ1 and VOZ2 repress *Hpa*-induced expression of ABA response genes, and this effect is augmented during BABA-IR against *Hpa*.

## Discussion

The resistance-inducing activities of BABA have been known for decades (Jakab et al., 2001; Cohen et al., 2016). However, the biological relevance of this resistance response has long remained unclear. Recently, Thevenet et al. (2017) reported that plant stress exposure induces endogenous BABA accumulation. Together with the discovery that the AspRS protein IBI1 acts as an *in planta* receptor of BABA (Luna et al., 2014; Buswell et al., 2018), these recent results strongly suggest that BABA acts as an endogenous immune signal in plants. Other studies have shown that BABA primes various immune responses, including SA- and NPR1-dependent defenses (Zimmerli et al., 2000; Ton and Mauch-Mani, 2004; Ton et al., 2005; Van der Ent et al., 2009; Pastor et al., 2013), as well as early-acting cell wall-localized defenses, such as apoplastic reactive oxygen species accumulation and callose deposition that are controlled by ABA signaling (Ton and Mauch-Mani, 2004; Ton et al., 2005; Flors et al., 2008). However, the immediate signaling steps by which the BABA-bound IBI1 mediates augmented defense expression, as well as the signaling role of ABA therein, have remained unresolved. Our study has identified two IBI1-interacting TFs, VOZ1 and VOZ2, which contribute to BABA-IR by mediating augmented expression of early-acting callose-associated defense at the cell wall (Figures 3 and 4). Both TFs are transcriptionally inducible by ABA but repress pathogen-induced ABA signaling during BABA-IR (Figure 6). Thus, our study has identified the VOZ TFs as novel signaling components in IBI1- and ABA-dependent expression of cell wall defense.

**Figure 7:**
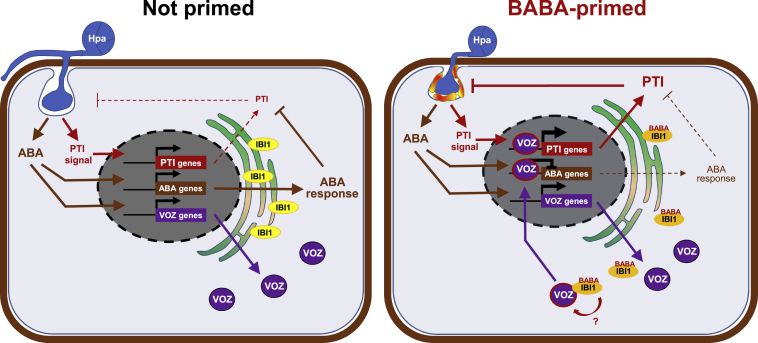
A Proposed Model Deciphering VOZ-Dependent Priming of Plant Immunity by BABA. Binding of BABA to its ER-localized receptor IBI1 primes this protein for pathogen-induced translocation to the cytoplasm (Luna et al., 2014). *Hpa* stimulates ABA signaling in the cell, which suppresses pattern-triggered immunity (PTI; Asai et al., 2014), but also increases *VOZ* gene expression. The combined induction of early-acting PTI genes and ABA-responsive abiotic tolerance genes (ABA genes) causes moderate levels of ER stress, which is sufficient to release IBI1 from the ER to the cytoplasm in BABA-primed cells. Cytosolic IBI1 subsequently interacts with the increased pool of VOZ transcription factors. Through yet unknown mechanisms, this interaction stimulates translocation of VOZ to the nucleus, where it represses expression of ABA genes while (directly or indirectly) increasing the expression of PTI genes (Nakai et al., 2013). The repressed ABA signaling in BABA-primed cells allows for augmented PTI signaling and resistance against *Hpa*, including callose-associated defense (Zimmerli et al., 2000; Ton et al., 2005; Schwarzenbacher et al., 2014). In unprimed cells, IBI1 is less responsive to *Hpa*-induced cytoplasmic translocation (Luna et al., 2014), which delays the IBI1/VOZ1/2-dependent signaling cascade. Consequently, there is no VOZ1/2-dependent repression of ABA signaling, resulting in basal resistance that is too weak to prevent infection. The model does not show the effects of the BABA-induced stress response, which may have a synergistic action on stress-related ABA signaling in the more sensitive *ibi1-1* mutant during *Hpa* infection (Luna et al., 2014).

The role of ABA in plant immunity has remained controversial (Ton et al., 2009; Berens et al., 2019). Our study points to a model that reconciles the controversy about ABA in plant immunity and explains the complex signaling role of ABA in BABA-IR (Figure 7). During BABA perception, the molecule binds to the L-Asp-binding domain of the IBI1 receptor (Luna et al., 2014; Buswell et al., 2018), which primes this protein for pathogen-induced translocation from the ER membrane to the cytoplasm (Luna et al., 2014). Upon *Hpa* infection, the pathogen stimulates ABA signaling as a virulence strategy to suppress pattern-triggered immunity (PTI; Asai et al., 2014), which elicits enhanced expression of *VOZ1* and *VOZ2* genes (Figure 6A and Supplemental Figure 4). Since induction of PTI genes causes ER stress (Wang et al., 2005; Moreno et al., 2012; Kørner et al., 2015), we propose that the *Hpa*-suppressed PTI during the early stages of infection is sufficient in BABA-primed cells to trigger moderate levels of ER stress and so allow for cytoplasmic translocation of IBI1, where it interacts with the ABA-induced pool of defense-regulatory VOZ1/2 TFs. Through yet unknown mechanisms, this interaction activates nuclear defense activity by the TFs (Figure 5B), which antagonizes ABA-dependent immune suppression by *Hpa* (Figure 6C) and facilitates augmented expression of callose-associated PTI (Figure 4C). In unprimed cells this IBI1/VOZ1/2-dependent signaling cascade is delayed, as IBI1 without BABA is less responsive to pathogen-induced cytoplasmic translocation (Luna et al., 2014), resulting in basal resistance that is too weak to prevent infection. It is worth noting that pathogen-induced production of endogenous BABA does not appear to have a significant contribution to basal resistance against *Hpa*, since control-treated wild-type plants displayed levels of *Hpa* colonization similar to those in *ibi1-1* plants (Figures 1B, 2D, 3B, and 5B). Thevenet et al. (2017) showed that BABA concentrations in *Arabidopsis* leaves increased significantly within 1 and 2 dpi with *P*. *syringae* and *Plectosphaerella cucumerina*, respectively, whereas endogenous BABA concentrations did not increase until 5 dpi with *Hpa*. Considering that BABA-augmented cell wall defense against *Hpa* is effective from 2–3 dpi, we conclude that *Hpa* employs specific effectors that suppress endogenous accumulation of BABA. This hypothesis also explains the relatively weak impact of the *ibi1-1* mutation on *Hpa*-induced gene expression in water-treated plants (Figure 1D).

The *voz1*/*2* mutant was not only affected in callose-associated cell wall defense (Figure 4C), but also showed a defect in BABA-induced priming of SA-induced expression of the *PR1* gene (Figure 4A). This latter result supports earlier findings that *voz1*/*2-2* plants are affected in SA-dependent basal acquired resistance and SAR against *P*. *syringae* pv. *tomato* DC3000 (Nakai et al., 2013). While SA-dependent defenses are important for basal resistance and SAR against *Hpa* (Delaney et al., 1994; Ton et al., 2002), the function of VOZ1/2 in priming of SA-induced gene expression did not have a major contribution to BABA-IR against *Hpa*, since SA non-accumulating *voz1*/*2-2 NahG* plants showed a stronger reduction in BABA-IR than *voz1*/*2* plants (Figure 4B). Unlike *ibi1-1* plants, SA non-accumulating *NahG voz1*/*2-2* plants still expressed a residual level of BABA-IR against *Hpa* (Figure 4B), indicating activity of additional VOZ1/2- and SA-independent defense layers, which act at different stages of infection than relatively early callose-associated defense and relatively late SA-dependent defense, respectively. This conclusion also supports the wider notion that BABA-IR is based on priming of PTI (Po-Wen et al., 2013), which constitutes a multitude of different defense layers that become active at different stages of infection (Zipfel and Robatzek, 2010; Bigeard et al., 2015).

Our Y2H screens for IBI1-interacting proteins identified 25 different protein candidates, including three high-confidence interactors which in other studies have been confirmed to be localized in the cytoplasm (Supplemental Figure 2 and Supplemental Table 3). It is, however, possible that IBI1 also interacts with proteins in other cellular compartments during BABA-IR. For instance, our confocal microscopy analyses of IBI1 and VOZ2 cannot exclude the possibility that the IBI1–VOZ complex transiently moves to the nucleus, where it interacts with other defense-regulatory proteins. In this regard, the D111/G-patch domain-containing protein is interesting, which showed a high-confidence interaction with IBI1 and is predicted to be targeted to the nucleus (Supplemental Table 3). The corresponding *At5G26610* gene was recently found to be translationally regulated during effector-triggered immunity and to regulate the hypersensitive cell death response (HR) (Yoo et al., 2020). In this context, it is possible that a transient interaction between the IBI1–VOZ complex and the D111/G-patch protein in the nucleus increases HR-related cell death and *Hpa* resistance. Furthermore, considering that the default localization of IBI1 is the ER (Luna et al., 2014), it is equally possible that IBI1 undergoes relevant interactions with ER-localized proteins. Of particular interest is the interaction with the ER-localized FAH2 protein (Supplemental Table 3). This fatty acid hydroxylase is required for the activity of Bax Inhibitor-1 (BI-1), which represses ER stress-related cell death (Watanabe and Lam, 2008; Nagano et al., 2012). Based on our model that PTI-related ER stress triggers IBI1 translocation to the cytosol (Figure 7), it is tempting to speculate that binding of BABA to IBI1 affects FAH2-dependent BI-1 activity, resulting in increased sensitivity to PTI-related ER stress and augmented IBI1 translocation to the cytoplasm during pathogen infection.

Although our study has identified a new regulatory module in plant acquired immunity, there are still unresolved questions about the role of ABA in BABA-IR against stresses other than *Hpa*. For example, the suppressive activity of IBI1 and VOZ1/2 on abiotic stress genes (Figures 2C, 6B, and 6C) seems difficult to reconcile with previous observations that BABA induces tolerance against drought and salt stress (Jakab et al., 2005). This could in part be explained by the fact that the signaling pathways controlling BABA-IR highly depend on the challenging stress (Zimmerli et al., 2000). For instance, while BABA primes for enhanced induction of the ABA-dependent *RAB18* gene after salt stress exposure (Jakab et al., 2005), this priming effect is not apparent after inoculation with *Alternaria brassicicola* or *P*. *cucumerina* (Ton and Mauch-Mani, 2004). Hence, the role of ABA in BABA-IR differs depending on the challenging stress, and it is possible that other IBI1-interacting proteins control these stress-specific augmented defenses in BABA-primed plants. Future research is needed to investigate how BABA manipulates the crosstalk between environmental signaling pathways. In the face of global climate change, a better understanding of the crosstalk between biotic and abiotic stress signaling is crucial for the breeding of crops that are able to cope simultaneously with drought, heat, pests, and diseases.

## Methods

### Plant Material and Growth Conditions

*A*. *thaliana* (ecotype Col-0) for bioassays and gene expression assays were grown on a mixture of Levington M3 soil and sand (2:1 [v/v]), at 8.5-h light (115–140 μmol/m^2^/s, 21°C) and 15.5-h darkness (19°C), and at 65%–80% relative humidity. *N*. *benthamiana* plants were grown at 15.5-h light (115–140 μmol/m^2^/s, 21°C) and 8.5-h darkness (19°C) in controlled growth chambers. Leaf infiltration assays were performed on 4- to 6-week-old plants (i.e., before plants developed flowers). Seeds of the *voz1-2 voz2-2* (*voz1*/*2-2*) mutant were kindly provided by M. Sato (Kyoto Prefectural University). *voz1-1 voz2-1* (*voz1*/*2-1*), *voz1*/*2-1 35S*:*GFP-VOZ2*, *voz1*/*2-1 35S*:*VOZ2-NLS*, and *voz1*/*2-1 35S*:*VOZ2-NES* seeds were kindly provided by T. Kohchi (Kyoto University). To generate homozygous *NahG voz1*/*2-2* lines, we crossed Col-0 *NahG* (line B15) reciprocally with the *voz1*/*2-2* double mutant. F2 progeny were genotyped by end-point PCR to select for individuals homozygous for the *voz1-2* and *voz2-2* T-DNA mutations and carrying the *NahG* gene. Two homozygous *voz1*/*2-2* F2 plants from each reciprocal cross with the strongest PCR band for *NahG* were selected for qPCR quantification of *NahG* DNA. Both plants displayed ~2-fold higher levels of *NahG* than their hemizygous F1 parents (Supplemental Figure 5A), suggesting that they were homozygous for *NahG*. Fourteen individual plants in the F3 progeny from one line (F3-1) were tested for the presence of the *NahG* gene (Supplemental Figure 5B), confirming that this line is no longer segregating for the *NahG* gene and thus homozygous for all three mutations/insertions. Seeds from this homozygous *NahG voz1*/*2-2* line were tested for BABA-IR against *Hpa* (Figure 4B).

### Induced Resistance Assays

Resistance against the biotrophic oomycete *H*. *arabidopsidis* (strain WACO9) was assessed microscopically in leaves of 3- to 4-week-old plants after soil-drenching plants with water or a racemic mixture of R/S-BABA (Sigma-Aldrich, #A44207), as described previously (Buswell et al., 2018). The BABA concentrations applied (5–10 mg/l) were sufficient to induce near complete levels of *Hpa* resistance, but low enough to prevent stress symptoms and growth reduction in wild-type plants (Col-0). To ensure that enough leaf material for the transcriptome analysis could be harvested, plants were 1 week older than the plants used in subsequent bioassays. At this older age, Col-0 plants require a higher BABA concentration to reach near complete levels of *Hpa* resistance. Accordingly, the final BABA concentrations in the soil were 10 mg/l for transcriptome analysis and 5 mg/l in all subsequent bioassays. Plants were inoculated with a suspension of *Hpa* conidiospores (10^5^ spores/ml) at 2 days after soil-drench treatment. Leaf samples were harvested at 5–7 dpi for trypan blue staining and scored for *Hpa* colonization by assigning individual leaves to four distinct colonization classes (Supplemental Figure 6): I = healthy leaf, no sporulation; II = hyphal growth, less than eight conidiophores; III = hyphal growth, and more than eight conidiophores; IV = extensive hyphal growth, conidiophores and oospores present. To assess the effectiveness of *Hpa*-elicited callose depositions, we collected samples at 2–3 dpi for aniline blue/calcofluor staining, as described previously (Ton et al., 2005). Stained leaves (>10 different leaves from independent plants) were analyzed by UV epifluorescence microscopy (Olympus BX51; light source: CoolLED pE-2; 330 nm wide band excitation filter, 400 nm LP emission filter, 400 nm dichromatic filter) and scored for numbers of callose-arrested versus unarrested germ tubes at the epidermal cell layer (Supplemental Figure 7). Statistical differences in class distributions of *Hpa* colonization and callose defense efficiency were determined by pairwise Fisher's exact tests with Benjamini–Hochberg false discovery rate (FDR) correction (Benjamini and Hochberg, 1995), using the R package “fifer” (https://cran.r-project.org/package=fifer).

### qRT–PCR Assays

For qRT–PCR quantification of gene expression, shoot samples from three to four biological replicates per treatment/genotype combination were snap-frozen in N_2_(l) and homogenized. Each biological replicate consisted of 4–10 seedlings/pot. Total RNA was extracted using the RNeasy Plant Mini Kit (Qiagen, cat. no. 74904), according to the manufacturer's protocol. RNA was treated with DNase (RQ-RNase free DNase, Promega, #M6101), and cDNA synthesis was performed from 800 ng of total RNA, using oligo(dT) primers with SuperScript III reverse transcriptase (Invitrogen, 18080085), according to the manufacturer's protocol. The cDNA was diluted 4–10× in nuclease-free water before qPCR in a RotorGene Q real-time PCR cycler (Qiagen; Q-Rex Software v1.0), using the RotorGene SYBR Green Kit (Qiagen, cat no. 204074) and gene-specific primer pairs at a final concentration of 250 nM (see Supplemental Table 4 for primer sequences). Specificity of primers was verified by dissociation melting curve analysis. For each primer pair, PCR efficiencies (1 + E) were determined by averaging machine-estimated PCR efficiencies of all replicate reactions per experiment. For each replicate sample, fold-change values in comparison with an arbitrary calibrator sample were calculated as (1 + E)^Ct1−Ct2^, where Ct (take-off cycle) was defined as the cycle at which the increase in fluorescence is 20% of the peak increase in fluorescence (Qiagen, 2012). Fold-change values were normalized to the averaged fold-change values of three housekeeping genes: GAPDH (At1g13440), UBC21 (At5g25760), and SAND family protein (At2g28390) (Czechowski et al., 2005). For each replicate sample, these corrected fold-change values were normalized to the averaged values of the control samples (e.g., water-treated and mock-inoculated Col-0 plants), as indicated in figure legends. Statistically significant differences in gene expression were determined by Student's *t*-tests or ANOVA (*n* = 3–4), as indicated in the figure legends.

### Yeast Two-Hybrid Analysis

The *IBI1* coding sequence (amino acids 1–558) (At4g31180; NCBI reference NM_119268.4) without STOP codon was PCR-amplified from pENTR-IBI1 and cloned either into pB29 as an N-terminal fusion to LexA DNA-binding domain (IBI1-LexA; first screen), or into pB43 as an N-terminal fusion to the Gal4 DNA-binding domain (IBI1-Gal4; second screen). The LexA construct was used as a bait to screen a random-primed cDNA library from etiolating *Arabidopsis* seedlings; the Gal4 construct was used as a bait to screen the dT-primed Universal Arabidopsis Normalized cDNA library (Clontech, #630487). The normalized cDNA was cloned into pGADT7-RecAB, resulting in a collection of Y2H prey plasmids encoding the prey fusion proteins (N-NLSSV40-GAL4-AD-HA-tag-prey-C). The pB29, pB43, and pP6 plasmids were derived from the pBTM116 (Vojtek and Hollenberg, 1995; Béranger et al., 1997), pAS2ΔΔ (Fromont-Racine et al., 1997), and pGAD.GH (Bartel and Fields, 1995) plasmids, respectively. Lack of toxicity and auto-activating activity by the IBI1-GAL4-DBD fusion protein was confirmed in initial test runs without 3-aminotriazol in the selection medium. For the first screen with the LexA bait construct, 72M clones (7-fold the complexity of the library) were screened, using a mating approach between YHGX13 (Y187 ade2-101::loxP-kanMX-loxP, matα) and L40ΔGal4 (mata) strains (Fromont-Racine et al., 1997), from which 30 His^+^ colonies were selected on medium lacking Leu, Trp, and His (−LTH). For the second screen with the Gal4 bait construct, 79.8M clones (7-fold the complexity of the library) were screened, using a mating approach between YHGX13 and CG1945 (mata) yeast strains (Fromont-Racine et al., 1997), from which 233 His^+^ colonies were selected on −LTH medium. Prey fragments of positive clones were amplified by PCR and sequenced at their 5′ and 3′ junctions. Sequences were blasted against the GenBank database (NCBI). A Predicted Biological Score (PBS) was attributed to each interaction, taking into account the redundancy and independency of prey fragments, the distribution of reading frames and stop codons in overlapping fragments, and interactions found in previous screens with the same library (Formstecher et al., 2005). PBS values ranged from A (highest confidence) to F (domains that have previously been confirmed as false positives), and have been demonstrated to correlate positively with biological significance of protein interactions (Rain et al., 2001; Wojcik et al., 2002).

### Bimolecular Fluorescence Complementation Assays

Coding sequences (CDSs; without stop codon) of the respective genes were amplified from *Arabidopsis* Col-0 cDNA using Phusion High-Fidelity DNA Polymerase (New England Biolabs, #M0530L) and cloned into pENTR plasmids (Invitrogen). Expression vectors were then created by Gateway cloning the CDSs of the putative interaction partners into the Gateway-compatible BiFC vectors pEarleygate201-YN (pEG201-YN) and pEarleygate202-YC (pEG202-YC). Leaves of 4- to 6-week-old *N*. *benthamiana* were infiltrated with *A*. *tumefaciens* (strain GV3101::pMP90) cell suspensions (OD_600_ = 1.0) carrying the appropriate recombinant plasmids. At 2–3 days after infiltration, interactions were assessed by analyzing fluorescence in three leaf discs (6 mm diameter) from at least two individually infiltrated leaves on an Olympus BX51 fluorescence microscope (light source: CoolLED pE-2; 460–490 nm wide band excitation filter, 510–550 nm emission filter, 505 nm dichromatic filter).

### Transcriptome Analysis

Three-week-old Col-0 and *ibi1-1* plants were cultivated, treated, and inoculated as described above. Three biological replicates (each consisting of pooled leaves from ~20 plants from one pot) were collected at 2 dpi and snap-frozen in N_2_(l) for total RNA extraction. RNA hybridization to Arabidopsis Gene ST 1.1 arrays (median 22 probes/gene, Affymetrix, #901913) was performed at the Nottingham Arabidopsis Stock Center. Microarray data were extracted, quality-checked, and normalized by robust multiarray average (RMA), using R Bioconductor software (version 3.2; Huber et al., 2015). Prior to normalization, an image of each microarray was produced using the package “affy” (version 1.48.0; Gautier et al., 2004) to verify absence of bubbles and smears. Normalization was performed by the RMA algorithm in package “oligo” (version 1.34.2; Carvalho and Irizarry, 2010), which implements background correction, log_2_ transformation, and quantile normalization of the raw hybridization values, thereby assigning identical statistical properties to the distribution of hybridization signals from each microarray. A linear model was fit to the normalized data to obtain an expression value for each probe set (Irizarry et al., 2003). Annotations of probe sets and transcripts were retrieved from the NetAffx database (Liu et al., 2003), using the “oligo” package. PCA at the transcript level across all 24 microarrays was performed, using the package “arrayQualityMetrics” (Kauffmann et al., 2009). DEGs between Col-0 and *ibi1-1* at each experimental condition were selected by pairwise comparisons of normalized hybridization values, using the “limma” package (linear mode + Benjamini–Hochberg FDR; *q* < 0.01; no cut-off value for fold change applied). Hierarchical clustering of DEGs was performed using the software MeV version 4.9.0 (Saeed et al., 2006), after row-normalization of gene expression values to the average across all samples. GO-term analysis of DEGs was performed using the web tool Gorilla (Eden et al., 2009; http://cbl-gorilla.cs.technion.ac.il/; accessed 11/02/2015).

### Microscopy Analysis of GFP-VOZ2

Microscopy analysis of GFP-VOZ2 subcellular localization was examined by epifluorescence microscopy and confocal microscopy of *voz1*/*2-1* plants overexpressing *GFP-VOZ2* (*p35S*:*GFP-VOZ2*; Yasui et al., 2012) Leaves for epifluorescence microscopy analysis were harvested at 4 dpi for DAPI staining, as described by Borg et al. (2019). In brief, samples were incubated in DAPI solution (1.5 μg/ml) for 15 min and washed with water, and individual leaves were mounted on microscope slides. Images were taken with a Leica DM6 B upright microscope (light source: CoolLED pE-2; GFP: 470/40 nm excitation filter, 525/50 nm emission filter, 495 nm dichroic filter; DAPI: 350/50 nm excitation filter, 460/50 nm BP emission filter, 400 nm LP dichroic filter), using Leica LAS X software. For confocal laser scanning microscopy, plants were harvested at 2 dpi. Images were taken in unprocessed intact leaves using an Olympus FV1000 confocal laser scanning microscope (excitation, 488 nm argon laser; emission filter, 510–550 nm).

## Funding

This work was supported by a grant from the 10.13039/501100000781European Research Council (ERC; no. 309944, “*Prime-A-Plant*”) to J.T., a Research Leadership Award from the 10.13039/501100000275Leverhulme Trust (no. RL-2012-042) to J.T., a 10.13039/501100000268BBSRC-IPA grant to J.T. (BB/P006698/1) and supplementary grant from Enza Zaden to J.T., and an 10.13039/501100000781ERC-PoC grant to J.T. (no. 824985, “*ChemPrime”*).

## Author Contributions

J.T. conceived the project; R.E.S. and J.T. designed and supervised experiments; R.E.S., G.W., E.G., P.J., E.L., and J.T. performed bioassays; R.E.S., J.S., and E.L. analyzed the microarray data; R.E.S. and J.T. wrote the manuscript. All authors reviewed and approved the final manuscript.

## Accession Numbers

Microarray data have been deposited at EMBL (E-MTAB-8720). Sequence data from this article can be found in The Arabidopsis Information Resource data library (www.arabidopsis.org) under the following accession numbers: TAIR: At3g41180 (IBI1); At1g28520 (VOZ1); At2g42400 (VOZ2); At4g26080 (ABI1); At5g57050 (ABI2); At5g59220 (HAI1); At1g07430 (HAI2); At1g52890 (NAC019); At5g51990 (CBF4); At2g14610 (PR1).

## Supplemental Information

Document S1. Supplemental Figures 1–7

Supplemental Table 1. Differentially Expressed Genes in Col-0 Leaves at 2 Days after Mock/*Hpa* Inoculation Shown are gene expression values obtained from Affymetrix GeneChip ST1.1 microarray hybridization after robust multiarray average processing (log_2_ transformation, normalization, and fitting to gene-wise linear models). Differential expression between *Hpa*- and mock-inoculated Col-0 was determined using the R package “limma” (linear model + Benjamini–Hochberg FDR; *q* < 0.01; no cut-off value for fold change applied).

Supplemental Table 2. Differentially Expressed Genes between Leaves of Col-0 and *ibi1-1* Plants under the Four Different Experimental Conditions Hybridization signals from Affymetrix GeneChip ST1.1 microarrays were normalized and fitted to gene-wise linear models. Differentially expressed genes (DEGs) between Col-0 and *ibi1-1* were selected under each experimental condition, using the R package “limma” (Linear model + FDR; *q* < 0.01). The expression ratio is calculated as the difference between the means of the log_2_-transformed values. **(A)** All DEGs between leaves of Col-0 and *ibi1-1* plants under the four different treatments. **(B)** Subset of 143 defense-related DEGs between leaves of Col-0 and *ibi1-1* plants, which differ in response to BABA + *Hpa* only. Genes found in cluster 1 or cluster 2 in Figure 2C are indicated in yellow and purple, respectively. **(C)** Subset of 136 stress-related DEGs between leaves of Col-0 and *ibi1-1* plants, which differ in response to BABA + mock only.

Supplemental Table 3. Putative Protein Interactors of IBI1 Identified via Two Independent Yeast Two-Hybrid Screens Proteins identified in the Y2H screen against IBI1 are listed according to their biological confidence score (AB = high confidence; D = medium confidence; see Methods). Since the prey library consisted of cDNA extracted from a mix of different tissues, not all IBI1 interactors are expressed in the leaves, which is where BABA-primed defense expression against *Hpa* takes place. Out of the 25 putative interactors, 15 show robust expression in the leaves (according to the eFP Browser; expression signal >100), of which three were high-confidence interactors that are localized in the cytoplasm.

Supplemental Table 4. List of DNA Primers Used for qRT–PCR, Cloning, and Genotyping

Document S2. Article plus Supplemental Information
