# Supplementary material for: The IBI1 Receptor of β-Aminobutyric Acid Interacts with VOZ Transcription Factors to Regulate Abscisic Acid Signaling and Callose-Associated Defense
Source: Mol Plant. 2020 Oct 5;13(10):1455–69. doi: 10.1016/j.molp.2020.07.010 (PMC7550849; doi:10.1016/j.molp.2020.07.010)
Supplement: Document S1. Supplemental Figures 1–7 [file mmc1.pdf]

Molecular Plant, Volume *13*

Supplemental Information

The IBI1 Receptor of β-Aminobutyric Acid Interacts with VOZ Transcription Factors to Regulate Abscisic Acid Signaling and Callose-Associated Defense

Roland E. Schwarzenbacher, Grace Wardell, Joost Stassen, Emily Guest, Peijun Zhang, Estrella Luna, and Jurriaan Ton

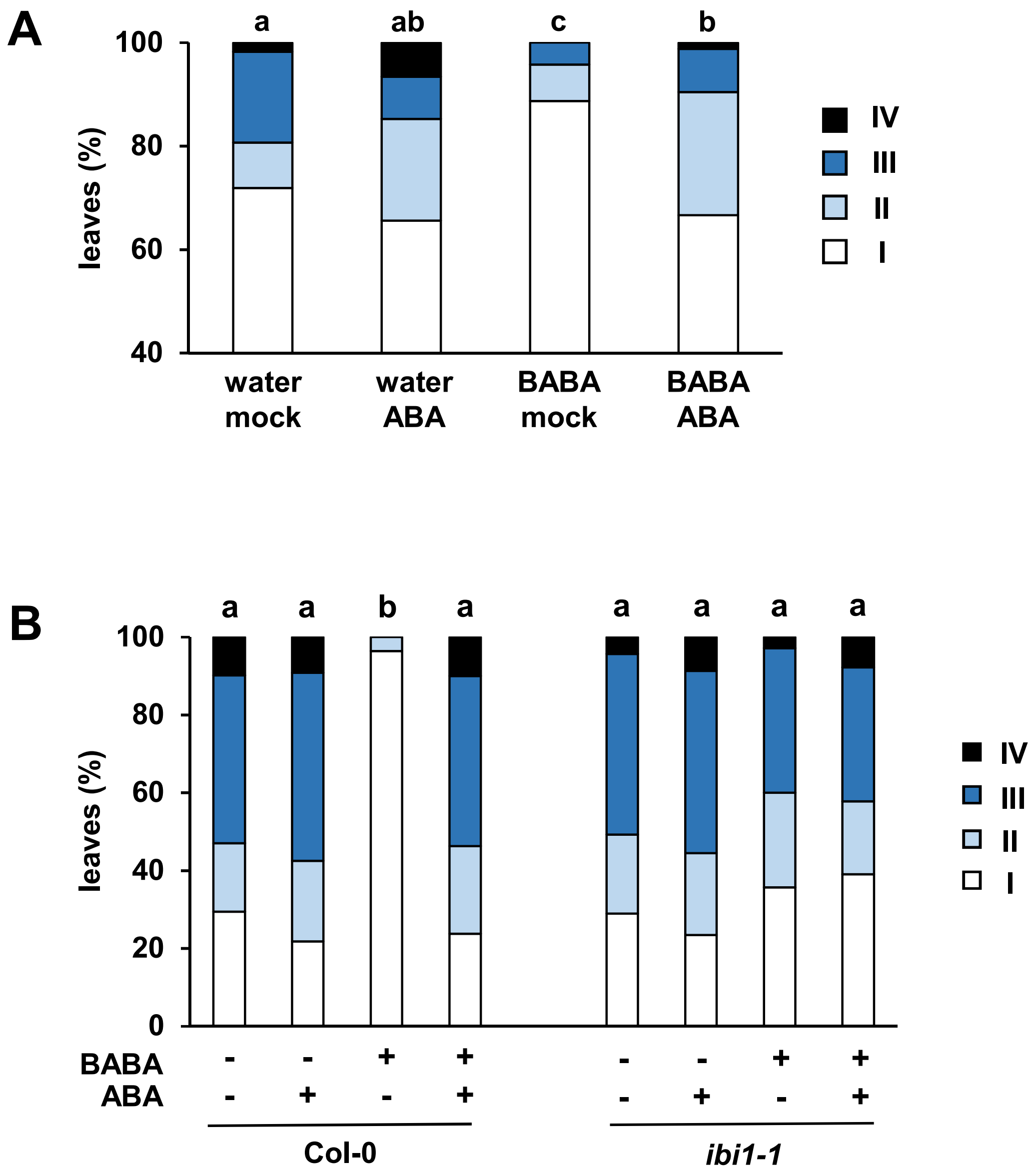

Supplemental Figure S1. The effect of ABA on BABA-induced resistance against *Hpa*. (**A**) Application of 100 mM ABA suppresses BABA-IR against *Hpa*. Two-week old Col-0 plants were pre-treated with water or 5 mg/L BABA. At 24 h after pre-treatment, plants were sprayed with ABA (100 µM + 0.01% silwet) or mock solution (0.5% EtOH + 0.01% silwet). At 48 h after pre-treatment, plants were challenged with *Hpa*. Disease progression was assessed at 5 dpi by assigning trypan blue-stained leaves to four different colonisation classes (Supplemental Figure S6). Different letters indicate statistically significant differences in class distribution between samples (Fisher's exact tests + FDR; $p<0.05$; n=57–84 leaves). (**B**) Application of ABA prior to BABA treatment suppresses BABA-induced resistance. Two-week old Col-0 and *ibi1-1* plants were sprayed with ABA (10 µM + 0.01% silwet) or mock solution (0.5% EtOH + 0.01% silwet). 24 hours after ABA treatment, plants were soil-drenched with 5 mg/L BABA or water. At 24 h after BABA treatment, plants were challenged with *Hpa*. Disease progression was assessed at 5 dpi by assigning trypan blue-stained leaves to four different colonisation classes (Supplemental Figure S6). Different letters indicate statistically significant differences in class distribution between samples (Fisher's exact tests + FDR; $p<0.05$; n=51-87 leaves).

A

screen	bait	library (cDNA)	all interactors	expressed in leaves	localised in cytoplasm	high confidence interactors
1	N-terminal IBI1 to LexA DNA-binding domain	shoot tissue of etiolated seedlings	3	1	1	1 (IBI1)
2	N-terminal IBI1 to Gal4 DNA-binding domain	normalised mix from different tissues	22	14	3	3 (IBI1, VOZ1 & VOZ2)

B

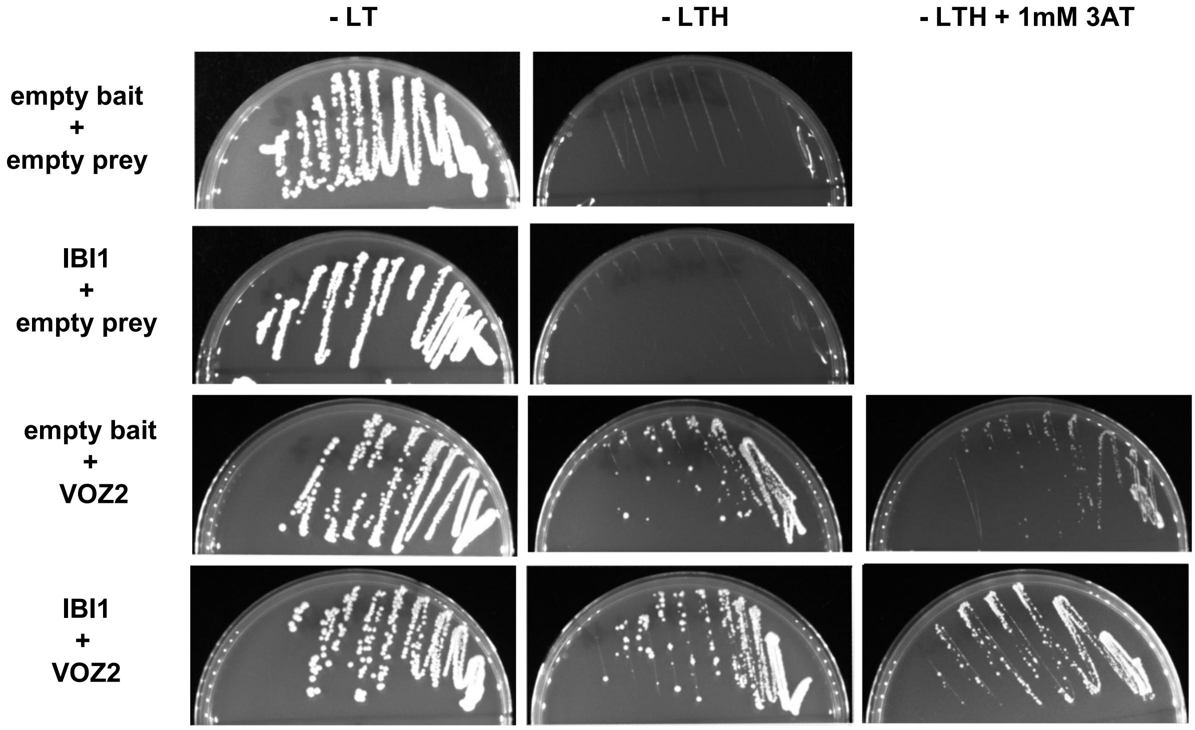

Supplemental Figure S2. Yeast two-hybrid analysis of putative IBI1 interactors. (**A**) Schematic diagram of the two yeast two-hybrid screens and selection of putative IBI1 interactors contributing to BABA-induced resistance. Expression in leaves was determined using the AtGenExpress visualisation tool (expression>100). Subcellular localisation was determined using the SUBA4 database (Hooper et al., 2017). (**B**) Yeast two-hybrid interaction between a positive VOZ2 clone from the primary screen and IBI1. Shown for all combinations (with and without IBI1-bait and VOZ2-prey) are agar plates containing drop-out medium without leucine and tryptophan (-LT), drop-out medium without leucine, tryptophan and histidine (-LTH), and dropout medium -LTH plus 1 mM 3-amino-1,2,3-triazole (3-AT) to counter weak auto-activation of the HIS3 reporter gene by VOZ2.

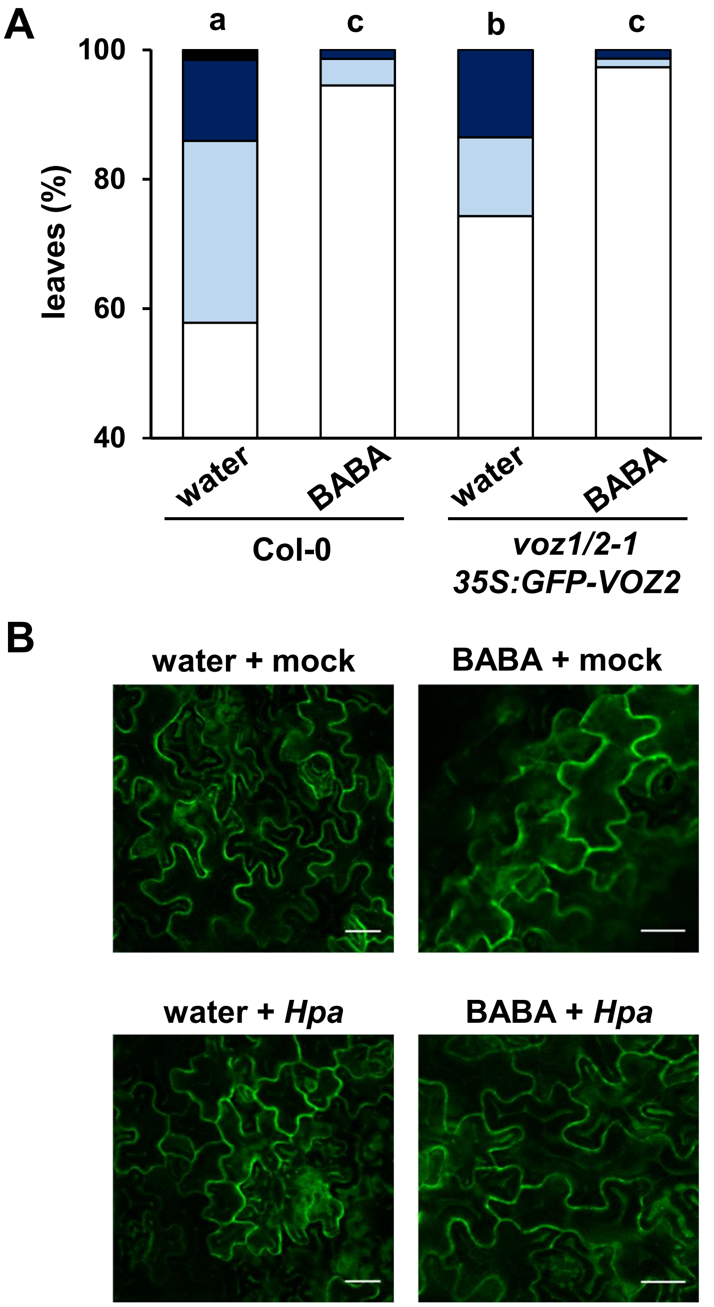

Supplemental Figure S3. Characterisation of transgenic *voz1/2-1* plants over-expressing *GFP-VOZ2*. (A) Over-expression of *GFP-VOZ2* restores BABA-IR against *Hpa* in the *voz1/2-1* double mutant. Two-week old Col-0 and *voz1/2-1 35S:GFP-VOZ2* plants were soil-drenched treated with water or 5 mg/L BABA and inoculated with *Hpa* 2 days later. Disease progression was analysed in trypan blue-stained leaves at 5 dpi by assigning leaves to different *Hpa* colonisation classes (Supplemental Figure S6). Different letters denote significant differences in class distributions (Fisher's Exact tests + FDR; $p<0.05$; n=60-100 leaves). (**B**) Confocal laser scanning microscopy of GFP-VOZ2 in epidermal leaf cells of *voz1/2-1 35S:GFP-VOZ2* shows that VOZ2 is predominantly localised in the cytoplasm under the four experimental conditions (water+mock, BABA+mock, water+*Hpa* and BABA+*Hpa*). Two-week old plants were soil-drenched with 5 mg/L BABA or water and challenge-inoculated with *Hpa* or water (mock) 2 days later. Leaves were analysed at 2 dpi. Scale bars = 50 mm.

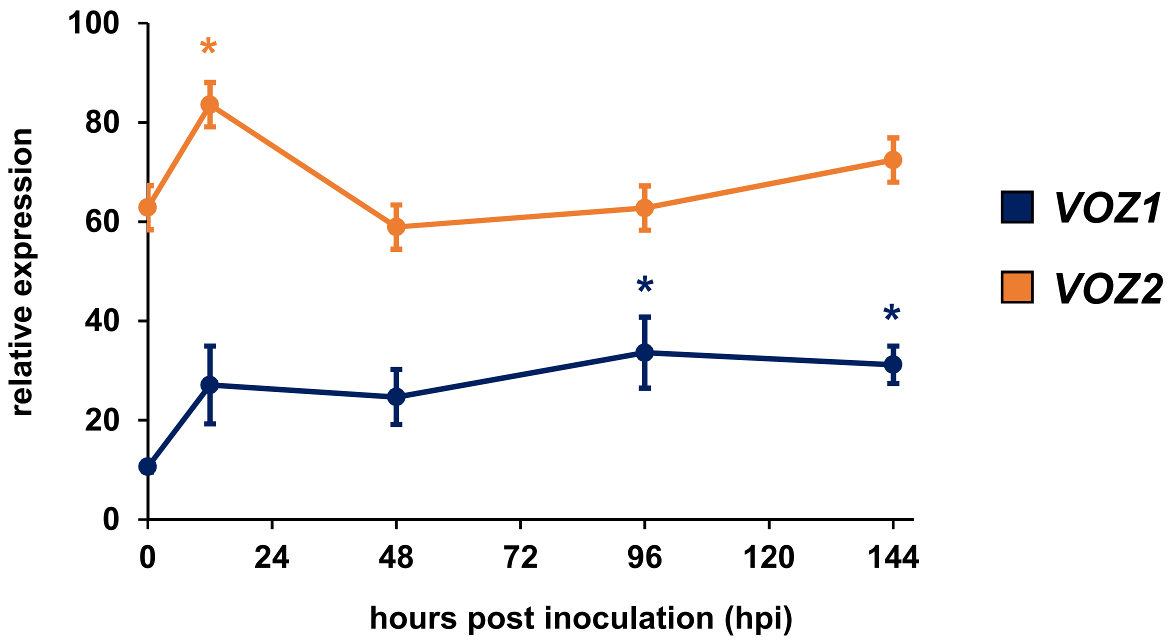

Supplemental Figure S4. Relative expression of *VOZ1* and *VOZ2* in the *rpp4* mutant after inoculation with *Hpa* Emwa1. Gene expression data were obtained from a publicly available transcriptome time-course experiment by Wang *et al.* (2011). Asterisks indicate statistically significant differences in relative expression compared to the baseline expression level at 0 hpi (Student's t-test; p<0.05).

A

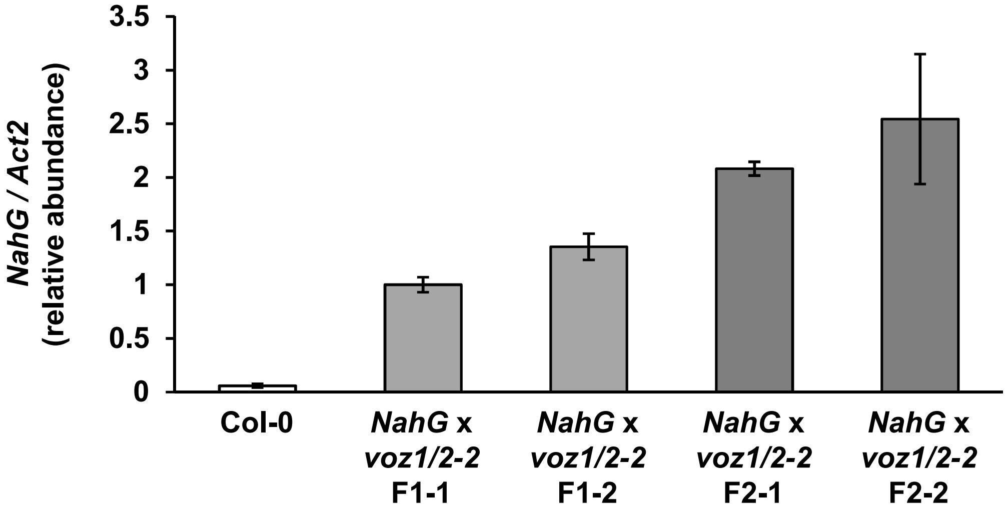

B

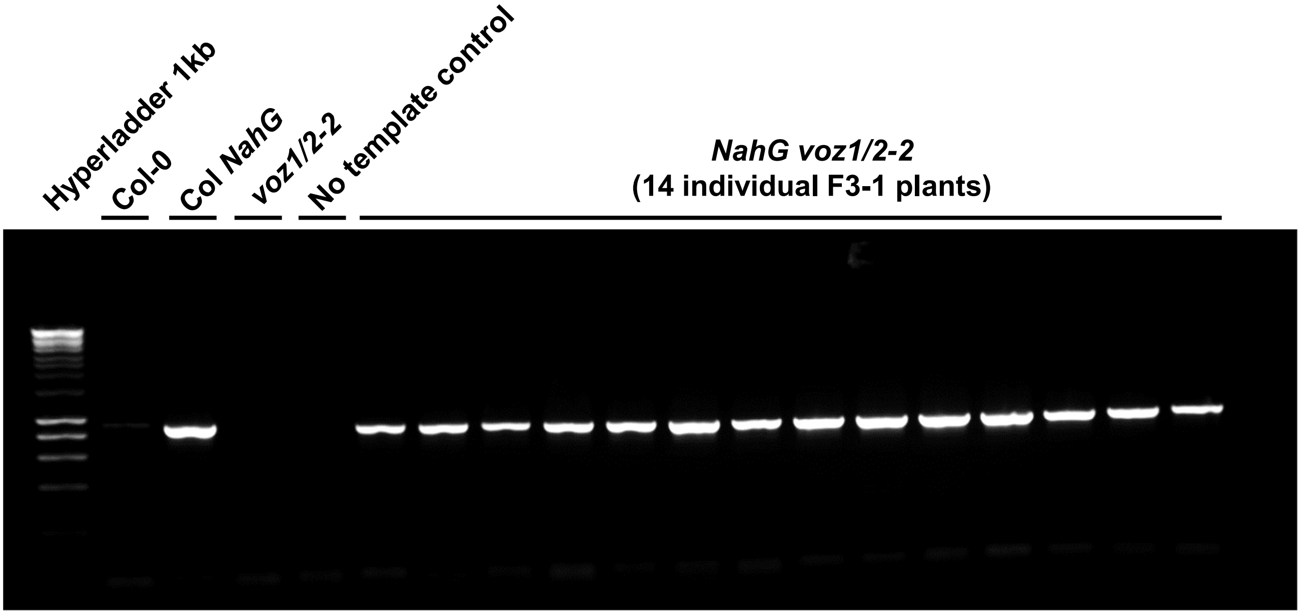

Supplemental Figure S5. Genotypic selection of NahG *voz1/2-2* plants. (**A**) qPCR quantification of *NahG* DNA in genomic DNA from Col-0, two F1 plants, and two F2 plants of the *NahG x voz1/2-2* cross. The relative abundance of *NahG* in the selected F2 plants is roughly 2-fold increased over their F1 parent plants, which is consistent with their suspected homozygosity for the *NahG* transgene. (**B**) Genotyping by end-point PCR of 14 F3 individuals from the F2-1 parent confirms homozygosity for the *NahG* transgene in this line. Primers used: NahG Fwd + NahG Rev (see Suppl. Table S4).

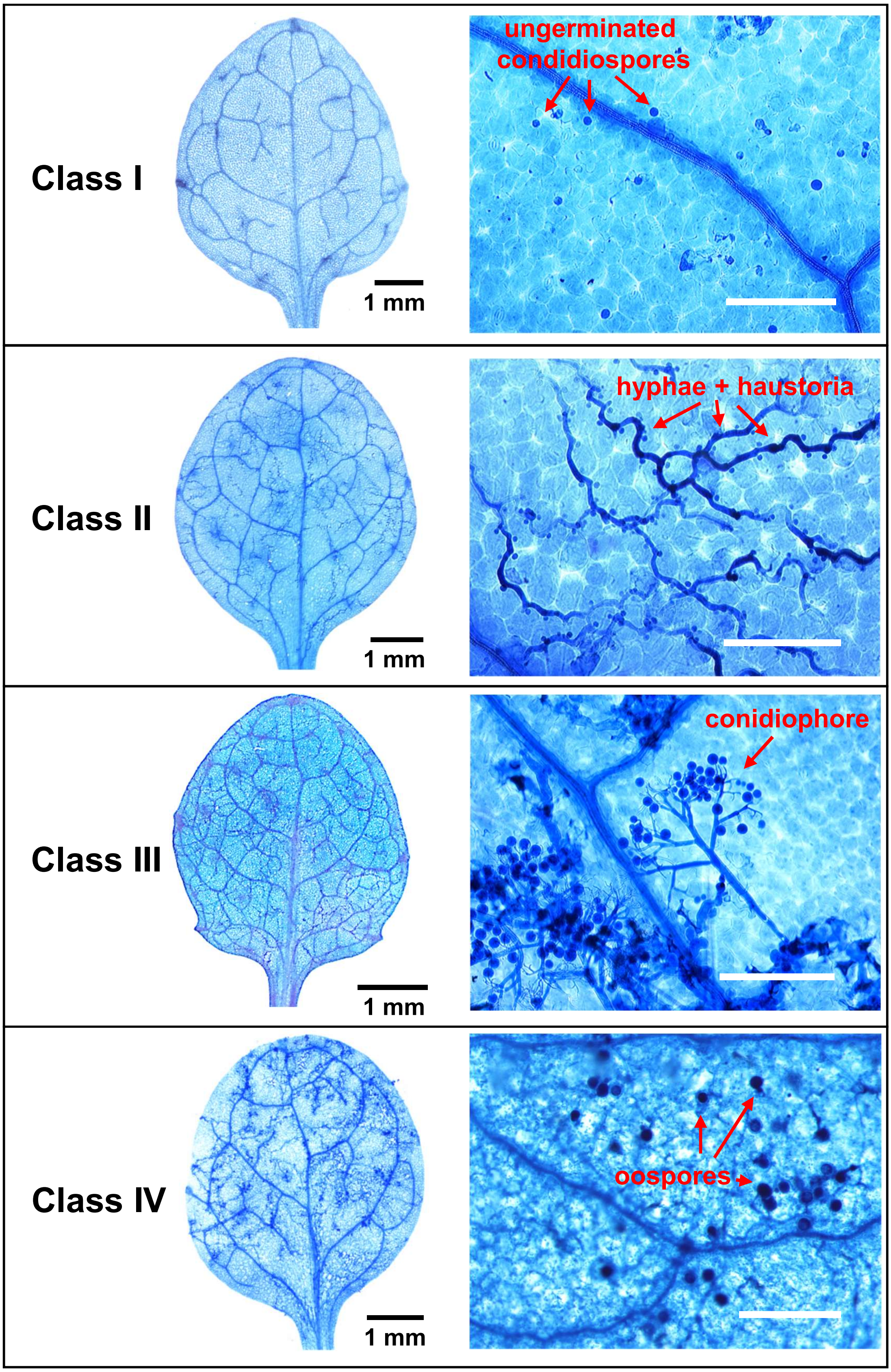

Supplemental Figure S6. Quantification of leaf colonisation by *Hpa*. Individual trypan blue-stained leaves of Arabidopsis seedlings are categorised across four different *Hpa* colonisation classes at 5-7 days post inoculation. The classification is based on oomycete structures that mark the stage of infection. Class I: healthy leaf, no hyphal colonisation by the conidiospores originating from the inoculum. Class II: limited hyphal colonisation and maximum 8 conidiophores/leaf. Class III: extensive hyphal colonisation with more than 8 conidiophores/leaf. Class IV: extensive hyphal growth, tissue collapse and presence of oospores. Images on the left are whole-leaf examples; images on the right are higher magnification to illustrate distinctive developmental structures of *Hpa*. White bars indicate scale bars (100 µm).

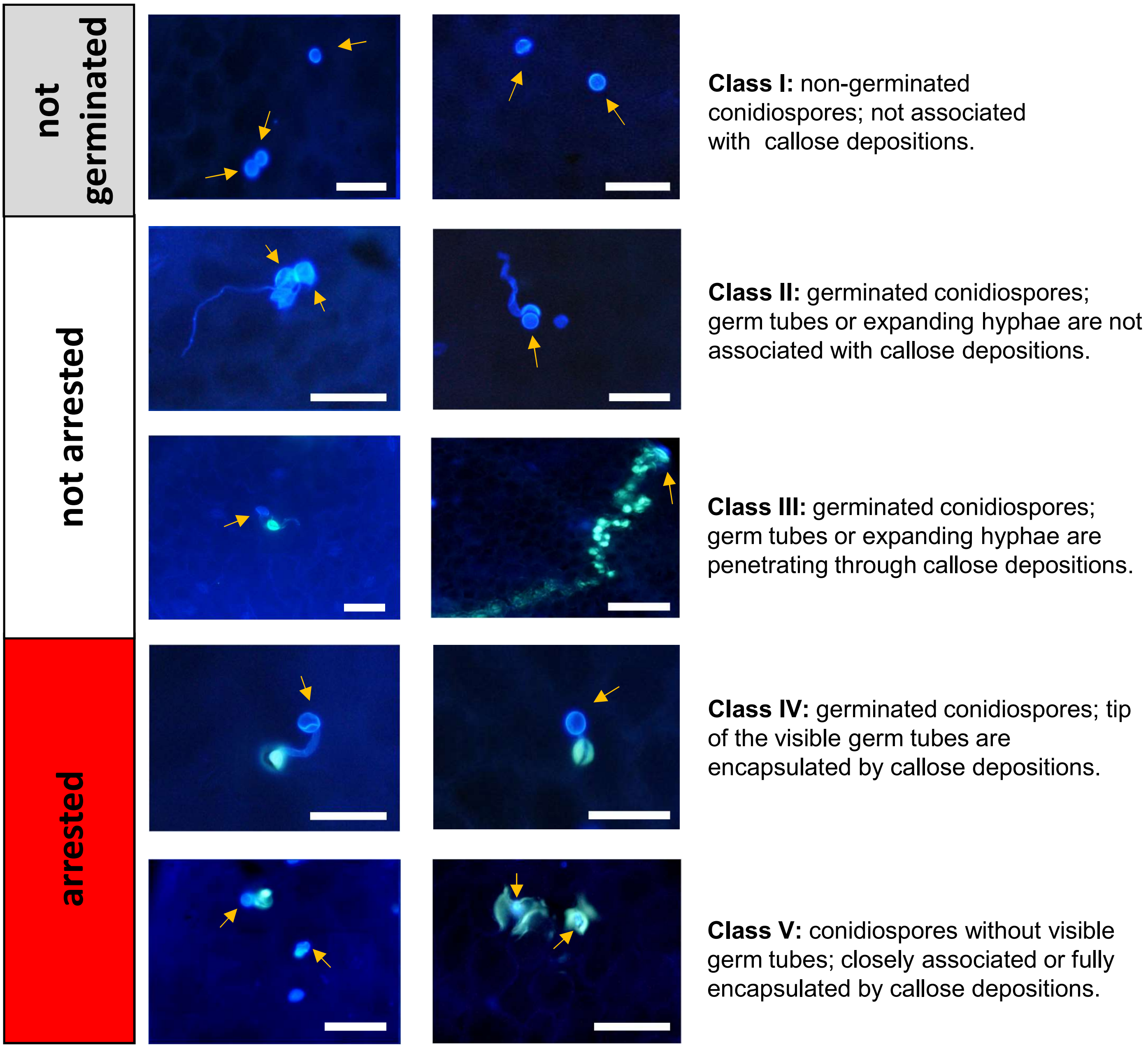

Supplemental Figure S7. Quantification of the effectiveness of callose-associated cell wall defence against *Hpa* infection. Shown are UV-epifluorescence photographs of *Hpa*-inoculated leaves after double-staining by calcofluor and aniline-blue. Cell wall defence was considered to be ineffective when the germ tubes/expanding hyphae were not associated with callose (class II), or when the germ tubes/expanding hyphae penetrated through the callose depositions (Class III). Cell wall defence was considered to be effective when the callose deposition encapsulated the proximal tip of the germ tube (class IV), or when it prevented germination of the conidiospore (class V). The effectiveness of cell wall defence was visualised by stacked bar graphs, showing relative frequencies (percentages) of non-arrested conidiospores (class II+III; white) versus arrested conidiospores (class IV+V; red). Conidiospores without germ tubes and associated callose (class I; grey) were considered non-germinated and not included in the analysis. Orange arrows indicate blue-fluorescent conidiospores by calcofluor staining. The callose is yellow/green-fluorescent by aniline-blue staining. White bars indicate scale bars (20 µm).
