# Supplementary material for: The IBI1 Receptor of β-Aminobutyric Acid Interacts with VOZ Transcription Factors to Regulate Abscisic Acid Signaling and Callose-Associated Defense
Source: Mol Plant. 2020 Oct 5;13(10):1455–69. doi: 10.1016/j.molp.2020.07.010 (PMC7550849; doi:10.1016/j.molp.2020.07.010)
Supplement: Supplemental Table 3. Putative Protein Interactors of IBI1 Identified via Two Independent Yeast Two-Hybrid Screens — Proteins identified in the Y2H screen against IBI1 are listed according to their biological confidence score (AB = high confidence; D = medium confidence; see Methods). Since the prey library consisted of cDNA extracted from a mix of different tissues, not all IBI1 interactors are expressed in the leaves, which is where BABA-primed defense expression against Hpa takes place. Out of the 25 putative interactors, 15 show robust expression in the leaves (according to the eFP Browser; expression signal >100), of which three were high-confidence interactors that are localized in the cytoplasm. [file mmc4.docx]

**Supplemental Table S3. Putative protein interactors of IBI1 identified via two independent yeast 2-hybrid (Y2H) screens.**

| **Confidence (PBS score)^a^** | **Gene** | **Gene name** | **Expressed in leaves^b^** | **sub-cellular localisation in cytoplasm^c^** | **Notes** |
| --- | --- | --- | --- | --- | --- |
| AB | At4g31180 | IBI1 | + | + | IBI1 auto-interaction; cytoplasmic subcellular localisation verified by Luna et al. (2014) |
| AB | At1g28520 | VOZ1 | + | + | Transcription factors involved in suppression of ABA-dependent abiotic stress tolerance; cytoplasmic localisation experimentally demonstrated by Yasui et al. (2012) |
| AB | At2g42400 | VOZ2 | + | + |  |
| AB | At3g47520 | plNAD-MDH | + | - | Plastidic NAD(H)-dependent malate dehydrogenase |
| AB | At1g26650 |  | + | - | 7 trans membrane-helix protein |
| AB | At5g26610 |  | + | - | D111/G-patch domain-containing protein |
| AB | At5g62850 | SWEET5 (VEX1) | +/- | - | Sucrose transporter, mainly expressed in floral tissue |
| AB | At3g01620 | PSG1 | - | - | Pollen-specific gene involved in pollen germination and pollen tube growth |
| D | At4g20870 | FAH2 | + | - | Fatty acid hydroxylase, required for the BI-1-mediated suppression of programmed cell death. Localised to the ER and required for the ER stress response regulator Bax-inhibitor-1 (Nagono et al. 2012). |
| D | At4g18800 | RAB11B | + | - | Located in ER and trans-Golgi network. Involved in root hair growth. |
| D | At4g38430 | ROPGEF1 | + | + | Interacts with Rop1, which is involved in pollen tube growth. Other Rop proteins are also involved in defence. |
| D | At3g11400 | EF3G1 | + | - | Translation initiation factor |
| D | At1g12810 | Proline-rich family protein | + | - | Expressed in leaves, but 5x higher expression in pollen |
| D | At1g52220 | CURT1C | + | - |  |
| D | At4g15040 | TCP4 | + | - | TCP family transcription factor, possible artefact? |
| D | At2g33150 | PED1, KAT2 | + | - | Organellar 3-ketoacyl-CoA thiolase, involved in fatty acid β-oxidation during germination and subsequent seedling growth. |
| D | At2g17710 |  | + | - | Much higher expression in seeds than leaves (~30x) |
| D | At2g44790 | UCLACYANIN2 | +/- | - |  |
| D | At1g48100 |  | +/- | - | Putative polygalacturonase, induced by wounding, but not *Hpa* |
| D | At4g25150 | HAD SUPERFAMILY IIIB ACID PHOSPHATASE | +/- | - | Mostly expressed in pollen |
| D | At1g32120 |  | +/- | - |  |
| D | At1g01460 | PIPK11 | - | - | Expressed in pollen |
| D | At2g39060 | SWEET9 | - | - |  |
| D | At1g10657 |  | - | - |  |
| D | GI:339773249 | Hypothetical mitochondrial protein | ? | - |  |

**^a^** Proteins identified in two separate Y2H screens against IBI1 are listed according to their confidence score (AB = high confidence; D = medium confidence). ^b^ Out of the 25 putative interactors, 15 show expression in the leaves according to publicly available gene expression data (Schmid et al., 2005; Klepikova et al. 2016). **^c^** Information based on experimental evidence and/or SUBA4 (Hooper et al., 2017).
